# Ferulic Acid as Building Block for the Lipase-Catalyzed Synthesis of Biobased Aromatic Polyesters

**DOI:** 10.3390/polym13213693

**Published:** 2021-10-27

**Authors:** Alfred Bazin, Luc Avérous, Eric Pollet

**Affiliations:** BioTeam/ICPEES-ECPM, UMR CNRS 7515, Université de Strasbourg, 25 Rue Becquerel, CEDEX 2, 67087 Strasbourg, France; alfred.bazin@etu.unistra.fr (A.B.); luc.averous@unistra.fr (L.A.)

**Keywords:** *Candida antarctica* lipase B, ferulic acid, semi-aromatic polyesters, biobased, enzymatic polymerization

## Abstract

Enzymatic synthesis of aromatic biobased polyesters is a recent and rapidly expanding research field. However, the direct lipase-catalyzed synthesis of polyesters from ferulic acid has not yet been reported. In this work, various ferulic-based monomers were considered for their capability to undergo CALB-catalyzed polymerization. After conversion into diesters of different lengths, the CALB-catalyzed polymerization of these monomers with 1,4-butanediol resulted in short oligomers with a DP_n_ up to 5. Hydrogenation of the double bond resulted in monomers allowing obtaining polyesters of higher molar masses with DP_n_ up to 58 and M_w_ up to 33,100 g·mol^−1^. These polyesters presented good thermal resistance up to 350 °C and T_g_ up to 7 °C. Reduction of the ferulic-based diesters into diols allowed preserving the double bond and synthesizing polyesters with a DP_n_ up to 19 and M_w_ up to 15,500 g·mol^−1^ and higher T_g_ (up to 21 °C). Thus, this study has shown that the monomer hydrogenation strategy proved to be the most promising route to achieve ferulic-based polyester chains of high DP_n_. This study also demonstrates for the first time that ferulic-based diols allow the synthesis of high T_g_ polyesters. Therefore, this is an important first step toward the synthesis of competitive biobased aromatic polyesters by enzymatic catalysis.

## 1. Introduction

The current environmental issues are directing research toward the production of greener materials through a more environmentally friendly processes. This trend is particularly important in the field of polyesters. Indeed, the vast majority of polyesters are fossil-based, often show poor biodegradability, and are synthesized through harsh reaction conditions [1].

For this reason, biobased polymers are gaining much attention. Indeed, such polymers are produced from a renewable feedstock [2,3] and can sometimes present good biodegradability [4]. However, similarly to their oil-based counterparts, these biobased polymers are also generally synthesized at high temperatures by employing potentially hazardous organometallic catalysts [5].

The lipase B from *Candida antarctica* (CALB) is an enzyme capable, in specific conditions, of catalyzing esterification and transesterification reactions. It is a versatile enzyme that accepts a wide variety of substrates [6]. Thus, CALB can be employed as a biocatalyst for the synthesis of various polyesters, replacing the organometallic catalyst and allowing milder reaction conditions [7,8]. As an example, our team studied the synthesis of various biobased aliphatic polyesters [9,10,11]. However, because of the superior thermal and mechanical properties of aromatic and semi-aromatic polyesters, their enzymatic synthesis is gaining attention [12,13]. As an example, the enzymatic synthesis of semi-aromatic furan-based polyesters has been studied [14,15,16,17], as these polymers represent a promising alternative to common oil-based aromatic polyesters such as polyethylene terephthalate [18]. However, furans are a particular case of C5 aromatic molecules. Therefore, we directed our research toward more classical C6 aromatic compounds as is the case for terephthalic acid. Several biobased compounds with C6 aromatic rings can be extracted from biomass and are promising candidates for biobased polymers synthesis, one of them being ferulic acid [19,20].

**Scheme 1:**
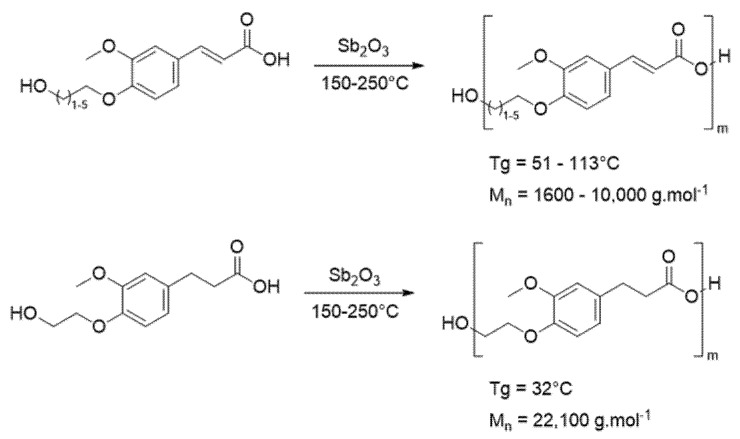
Representation of the synthesis of polyesters from ferulic derivatives of different lengths as well as with and without hydrogenation. Adapted from [29].

Ferulic acid can be extracted from various natural sources such as orange pulp, sugarcane bagasse, or corn and wheat bran [21]. This compound is present in hemicellulose as a crosslinker of the polysaccharide chains [22] and can also be produced from lignin [23]. Ferulic acid has been considered for potential uses as an additive in food and cosmetic products due to its interesting antioxidant and potential drug-like properties [24,25,26]. As a result of its rigid structure and adequate functionality, ferulic acid has been investigated for the synthesis of various polyesters with advanced properties. As an example, Thi et al. investigated the homopolymerization of coumaric derivatives, such as ferulic acid, catalyzed by an anhydride at temperatures up to 200 °C. The obtained polyesters showed interesting liquid–crystalline behavior but presented limited solubility in common organic solvents. Indeed, purely ferulic-based polymers result in very rigid materials with high glass transition and melting temperatures around 113 °C and 325 °C [27], respectively. The processability of these materials ends up hindered, and strategies of copolymerization as well as the modification of ferulic acid have been investigated to produce materials with more controlled and appropriate thermal properties [28]. Nguyen et al. [29] studied the polymerization of ferulic acid derivatives bearing hydroxyether moieties of various lengths, as represented in Scheme 1. Varying the length of the hydroxyether allowed fine-tuning the polymers’ thermal properties. The authors also showed the strong influence of the ferulic acid double bound on the polyester thermal properties by hydrogenating the ferulic derivatives. The same authors obtained interesting results on the copolymerization of these derivatives with cyclic monomers such as ε-caprolactone or l-lactide [30].

Recently, Kurt et al. [31] used hydrogenated derivatives of ferulic acid for the synthesis of aromatic polyesters catalyzed by zinc acetate and obtained M_n_ up to 8000 g·mol^−1^. Moreover, because of its therapeutic as well as anti-UV properties, native unmodified ferulic acid also allows the synthesis of material with innovative properties. As an example, Ouimet et al. produced a biobased copolyester from ferulic acid and diethyl adipate. Upon degradation, this polymer then allowed the controlled release of ferulic acid as an antibacterial and antioxidant agent [32]. Recently, Parthiban et al. used ferulic acid for the synthesis of UV-absorbing polymers. Pospiech et al. [33] showed that insertion of ferulic acid units in an aliphatic polyester significantly improved the material T_g_.

Enzymes such as CALB have been investigated by Pion et al. in processes for the synthesis of ferulic-based monomers subsequently used for the elaboration of various materials [34]. This method allowed the production of various polymers such as isocyanate-free poly(hydroxy)urethanes [35] and epoxy thermosets [36]. Polyesters were also synthesized with acyl chloride [37] or by ADMET [38]. These studies showed the great potential of ferulic acid for the synthesis of materials with tunable thermal and mechanical properties as well as antioxidant properties. However, the enzymatic catalysis was not used for the polymerization step. The polymerization of ferulic-based monomers is often performed under harsh thermal conditions that can be deleterious to the monomer integrity, leading to a coloration of the material. Moreover, the often toxic metal-based catalyst can remain in the final material, leading to environmental and health issues [39].

CALB has already been used for the synthesis of aromatic polyesters [40]. However, lipase-catalyzed polymerization of aromatic monomers often results in low molar mass polyesters [14,15]. Moreover, to the best of our knowledge, CALB and lipases in general have never been studied as biocatalysts for the polymerization of ferulic monomers.

In this study, ferulic acid was modified into new diesters of different lengths and polymerized with 1,4-butanediol using CALB as a biocatalyst. Then, two strategies were developed to enhance the reactivity of ferulic-based monomers in CALB-catalyzed polymerization and achieve higher molar masses: the hydrogenation and reduction of the monomer. Then, the structure and the properties of the final materials were investigated. The impact of the structure of the ferulic-based moieties first on the reactivity of the CALB and then on the thermal properties (glass transition, melting temperature, and degradation temperatures) of the final materials were assessed.

## 2. Materials and Methods

### 2.1. Materials

Lipase B from *Candida antarctica* immobilized on acrylic resin (activity measured to 11,000 PLU/g (propyl laurate units)) (CALB), deuterated chloroform (CDCl_3_), dibutyl aluminum hydride (DIBAL-H) solution in toluene (25 wt %), and palladium on activated charcoal (Pd/C) were supplied by Sigma-Aldrich (Saint-Louis, MO, USA). Diethyl adipate (DEA), potassium iodine, methyl 4-chlorobutyrate, and diphenyl ether were purchased from Acros Organics (Geel, Belgium). 1,4-butanediol (1,4-BDO), acetophenone, ferulic acid, and methyl chloroacetate were supplied by Alfa Aesar (Heysham, UK). Methanol and potassium carbonate were supplied by Fisher scientific (Illkirch-Graffenstaden, France). Chloroform was supplied by Carlo Erba (Emmendingen, Germany). CALB was dried at 25 °C for 24 h under vacuum before use. Other reactants were employed without further purification.

### 2.2. Characterization

A 400 MHz (Bruker, Wissembourg, France) spectrometer was used for ^1^H NMR analysis, and a Bruker 500 MHz spectrometer was used for 2D and ^13^C NMR analyses. The solvents employed were CDCl_3_ and DMSO-*d_6_*, depending on the solubility of the substrate. Calibration of the spectra was performed using the CDCl_3_ peak (δH = 7.26 ppm, δC = 77.16 ppm) and DMSO-*d6* peak (δH = 2.50 ppm, δC = 39.52 ppm).

Size Exclusion Chromatography (SEC) was performed to estimate the number average molar mass (M_n_), mass average molar mass (M_w_), and dispersity (Đ). The analyses were performed in THF at 40 °C in an Acquity-APC (Waters, Guyancourt, France) equipped with three columns (Acquity APC XT 450, 200 and 45Å 2.5 µm 4.6 × 150 mm). First, 10 µL of dissolved polymer were injected, and a flow of 0.6 mL/min was applied for the 11 min run. Refractive index (RI) detector and photodiode array (PDA) detector at 254 nm were used. A calibration curve with polystyrene (PS) standards was carried out for molar mass determination. The molar mass calculation was performed with data collected from the UV detector.

Differential scanning calorimetry (DSC) analyses were performed using a Q2000 DSC apparatus from TA Instrument (TA Instrument, New Castle, DE, USA) to estimate the polymers’ glass transition temperature (T_g_) as well as their melting temperature (T_m_) and crystallization temperature (T_c_), if applicable. Typically, a 1 to 3 mg sample in a sealed aluminum pan was treated following a three-step method: (1) fast heating to 180 °C (the sample was kept for 3 min at this high temperature to erase all thermal history), (2) cooling at 10 °C/min to −80 °C followed by an isotherm of 5 min, and (3) second heating at 10 °C/min to 180 °C. The characteristic temperatures were determined from this second heating scan. DSC was employed as well for the determination of the synthesized compounds melting points.

Thermogravimetric analysis (TGA) was performed to determine the thermal stability of the polymers. The measurements were conducted on a High-Res TGA Q5000 from TA Instrument (New Castle, DE, USA). Samples between 1 and 2 mg were heated at 10 °C/min from room temperature to 600 °C.

Infrared spectroscopy (IR) was performed with a Nicolet 380 Fourier transformed infrared spectrometer (Thermo Electron Corporation, Waltham, MA, USA) used in reflection mode and equipped with an ATR diamond module (ATR-FTIR). The spectra were collected at a resolution of 4 cm^−1^ and with 32 scans per run.

MALDI-TOF analyses were carried out on an AutoflexTM MALDI-TOF mass spectrometer (Bruker Daltonics GmbH, Bremen, Germany) used at a maximum accelerating potential of 20 kV in positive mode and operated in linear mode. The delay extraction was fixed at 560 ns, and the frequency of the laser (nitrogen 337 nm) was set at 5 Hz. The acquisition mass range was set to 1500–10,000 m/z with a matrix suppression deflection (cut off) set to 1500 m/z. The equipment was calibrated with ACTH 1-17 ([M+H]^+^ 2094.42), insulin ([M+H]^+^ 5732.52), ubiquitin I ([M+H]^+^ 8565.76), and myoglobin ([M+H]^+^ 16,952.31). Spectra were processed with flexAnalysis software (Version, Bruker Daltonics, Billerica, MA, USA). Sample preparation was performed with the dried droplet method using a mixture of 0.5 µL of sample with 0.5 µL of matrix solution dried at room temperature. The 2-[(2E)-3-(4-tert-butylphenyl)-2-methylprop-2-enylidene]malononitrile (DCTB) matrix was prepared at 20 mg/mL in dichloromethane.

### 2.3. Synthesis of Ferulic Diesters

Ferulic acid was dissolved in a large volume of methanol. A few drops of sulfuric acid were added as the catalyst. The solution was refluxed overnight. Then, the solution was neutralized by a few drops of saturated sodium carbonate solution. Afterwards, the solvent was evaporated, and the product was recovered in ethyl acetate and dried over MgSO_4_. The salts were extracted by filtration, and the solvent was evaporated. Methyl ferulate (**1**) was obtained as a clear yellow oil and used without further purification. (Yield: quantitative, IR: 3391 cm^−1^ (O-H), 2949 cm^−1^ (C-H), 1698 cm^−1^ (C=O ester), 1510 cm^−1^ (C=C Ar), 1262 cm^−1^ (C-O ester), ^1^H NMR (400 MHz, CDCl_3_) δ (in ppm): 7.62 (d, J = 15.9 Hz, 1H), 7.07 (dd, 1H), 7.03 (d, J = 1.9 Hz, 1H), 6.92 (d, J = 8.2 Hz, 1H), 6.29 (d, J = 15.9 Hz, 1H), 5.83 (brs, 1H), 3.93 (s, 3H), 3.80 (s, 3H)). ^13^C NMR (126 MHz, CDCl_3_) δ (in ppm): 167.81, 148.08, 146.87, 145.04, 126.93, 123.05, 115.12, 114.83, 109.47, 77.10, 55.95, 51.64, 30.93.

Then, the methyl ferulate (**1**) (4 g, 19.2 mmol, 1 eq.) was dissolved in acetonitrile (95 mL). Potassium carbonate (7.97 g, 57.63 mmol, 3 eq.) and potassium iodine (0.77 g, 4.61 mmol, 0.24 eq.) were added as catalyst. The corresponding chloroester (methyl chloroacetate or methylchlorobutyrate) was added (38.42 mmol, 2 eq.). Then, the solution was refluxed overnight. After cooling down, the solution was filtered to eliminate the undissolved catalyst. The solvent was evaporated under reduced pressure. The crude product was recovered in ethyl acetate and washed once with a saturated solution of sodium carbonate and twice with brine. The organic solution was dried over MgSO_4_, and the solvent was evaporated. Then, the product was recrystallized in a mixture of ethanol and water.

The methyl 4-(methyl ethanoate-oxy)-ferulate (**2a**) was obtained as white crystals (yield: 80%, melting point: 64 °C, IR (in cm^−1^): 2952 (C-H), 1735 (C=O ester), 1709 (C=O ester), 1517 (C=C Ar), 1268 (C-O ester), ^1^H NMR (400 MHz, CDCl_3_) δ (in ppm): 7.62 (d, J = 16.0 Hz, 1H), 7.12–7.02 (m, 2H), 6.78 (d, J = 8.8 Hz, 1H), 6.32 (d, J = 16.0 Hz, 1H), 4.73 (s, 2H), 3.91 (s, 3H), 3.80 (s, 6H)). ^13^C NMR (126 MHz, CDCl_3_) δ (in ppm): 169.02, 167.56, 149.70, 149.14, 144.49, 128.90, 122.09, 116.31, 113.52, 110.59, 77.06, 66.10, 56.00, 52.38, 51.70.

The methyl 4-(methyl butanoate-oxy)-ferulate (**2b**) was obtained as off-white needles (yield: 80%, melting point: 83 °C, IR (in cm^−1^): 2952 (C-H), 1699 (C=O ester), 1510 (C=C Ar), 1250 (C-O ester), ^1^H NMR (400 MHz, CDCl_3_) δ (in ppm) 7.62 (d, J = 15.9 Hz, 1H), 7.13–7.01 (m, 2H), 6.86 (d, J = 8.3 Hz, 1H), 6.30 (d, J = 16.0 Hz, 1H), 4.09 (t, J = 6.3 Hz, 2H), 3.88 (s, 3H), 3.79 (s, 3H), 3.68 (s, 3H), 2.54 (t, J = 7.2 Hz, 2H), 2.23–2.08 (m, 2H)). ^13^C NMR (126 MHz, CDCl_3_) δ (in ppm): 173.54, 167.69, 150.46, 149.60, 144.80, 127.54, 122.52, 115.53, 112.66, 110.22, 77.09, 67.79, 55.96, 51.68, 51.63, 30.41, 24.40.

### 2.4. Hydrogenation of the Ferulic Diesters

Pd/C (75 mg, 5 wt %) and the diester **2a** or **2b** (1.5 g, 5.1 mmol) were placed in a round-bottom flask and sealed with a septum. The powders were placed under argon, and ethanol (15 mL) was carefully added with a syringe. Then, hydrogen was bubbled for 15 min in the solution. Finally, the solution was stirred for 24 h with hydrogen overpressure. The solution was filtered through celite, and the solvent was evaporated under reduced pressure.

The methyl 3-(3-methoxy-4-(2-methoxy-2-oxoethoxy)phenyl)propanoate (**4a**) was obtained as a transparent oil (yield: 97%, IR (in cm^−1^): 2952 (C-H), 1731 (C=O ester), 1512 (C=C Ar), 1258 (C-O ester), ^1^H NMR (400 MHz, CDCl_3_) δ (in ppm): 6.80–6.65 (m, 3H), 4.67 (s, 2H), 3.87 (s, 3H), 3.79 (s, 3H), 3.67 (s, 3H), 2.90 (t, J = 7.5 Hz, 2H), 2.61 (t, J = 7.5 Hz, 2H)). ^13^C NMR (126 MHz, CDCl_3_) δ (in ppm) 173.32, 169.61, 149.57, 145.69, 135.08, 120.11, 114.56, 112.38, 77.13, 66.62, 55.88, 52.18, 51.64, 35.84, 30.61.

The methyl 4-(2-methoxy-4-(3-methoxy-3-oxopropyl)phenoxy)butanoate (**4b**) was obtained as a yellow oil (yield: 99%, IR (in cm^−1^): 2951 (C-H), 1731 (C=O ester), 1513 (C=C Ar), 1257 (C-O ester), ^1^H NMR (400 MHz, CDCl_3_) δ (in ppm): 6.80 (d, J = 7.9 Hz, 1H), 6.75–6.65 (m, 2H), 4.03 (t, J = 6.3 Hz, 2H), 3.84 (s, 3H), 3.67 (d, J = 3.6 Hz, 6H), 2.89 (t, J = 7.8 Hz, 2H), 2.61 (t, J = 7.5 Hz, 2H), 2.54 (t, J = 7.3 Hz, 2H), 2.13 (m, 2H)). ^13^C NMR (126 MHz, CDCl_3_) δ (in ppm): 173.72, 173.42, 149.50, 146.75, 133.63, 120.19, 113.62, 112.25, 77.09, 68.03, 55.94, 51.64, 51.62, 35.99, 30.62, 30.54, 24.60.

### 2.5. Synthesis of the Ferulic Diols

Ferulic diester **2a** or **2b** (1.5 g) was added to a three-neck flask equipped with a condenser and a dropping funnel. The flask was sealed with a septum and flushed with argon. Dry toluene (55 mL) was added. The solution was cooled at −78 °C, and a solution of DIBAL-H in toluene (27.1 mmol, 5 eq.) was added dropwise over 20 min. Then, the solution was left for 2 h at room temperature. Afterwards, the solution was cooled down at 0 °C, and ethyl acetate (20 mL) was carefully added. Then, a saturated solution of Rochel salts was added (30 mL) to complete the quenching of the reaction medium. The solution was left stirring overnight. The aqueous phase was washed 3 times with ethyl acetate. Then, the organic phase was washed once with brine and dried over MgSO_4_. Afterwards, the solvent was removed under reduced pressure.

The 4-(hydroxyethoxy)-coniferyl alcohol (**6a**) was obtained as a white powder (yield: 94%, melting point: 103 °C, IR (in cm^−1^): 3448 (O-H), 2932 (C-H), 1511 (C=C Ar), ^1^H NMR (400 MHz, DMSO-*d6*) δ (in ppm): 7.05 (s, 1H), 6.90 (s, 2H), 6.47 (d, J = 15.9 Hz, 1H), 6.26 (dt, J = 15.9, 5.3 Hz, 1H), 4.84 (t, J = 5.5 Hz, 1H), 4.81 (t, J = 5.4 Hz, 1H), 4.10 (t, J = 4.8 Hz, 2H), 3.96 (t, J = 5.1 Hz, 2H), 3.78 (s, 3H), 3.71 (q, J = 5.3 Hz, 2H)). ^13^C NMR (126 MHz, DMSO-*d6*) δ (in ppm): 149.51, 148.12, 130.43, 129.04, 129.00, 119.61, 113.49, 109.79, 70.63, 62.11, 60.06, 55.86, 39.98.

The 4-(hydroxybutoxy)-coniferyl alcohol (**6b**) was obtained as an off-white powder (yield: 98%, melting point: 87 °C, IR (in cm^−1^): 3273 (O-H), 2923 (C-H), 1512 (C=C Ar), ^1^H NMR (400 MHz, DMSO-*d6*) δ (in ppm): 7.04 (s, 1H), 6.88 (s, 2H), 6.46 (d, J = 15.9 Hz, 1H), 6.25 (dt, J = 15.9, 5.3 Hz, 1H), 4.80 (t, J = 5.4 Hz, 1H), 4.44 (t, J = 5.2 Hz, 1H), 4.10 (td, J = 5.3, 1.7 Hz, 2H), 3.94 (t, J = 6.5 Hz, 2H), 3.78 (s, 3H), 3.45 (q, J = 6.4 Hz, 2H), 1.85–1.67 (m, 2H), 1.64–1.50 (m, 2H)). ^13^C NMR (126 MHz, DMSO-*d6*) δ (in ppm): 149.54, 148.14, 130.30, 129.05, 128.94, 119.64, 113.42, 109.84, 68.59, 62.10, 60.90, 55.96, 39.99, 29.50, 26.02.

### 2.6. Enzymatic Polymerization

Each polyester was synthesized according to a procedure adapted from a previous study [17] (with optimal temperature, solvent, and concentrations) but varying the nature of the solvent, reaction time, and pressure when necessary or desired. Then, the obtained polymers were recovered with the same procedure as in the literature.

*From ferulic-based diesters (**3a-b** and **5a-b**):*

The ferulic-based diester (150 mg) and 1,4-BDO were placed in a Schlenck reactor in equimolar proportions. Then, diphenyl ether (300 wt % vs. the mass of the monomers) and CALB (20 wt % regarding the mass of the monomers) were added. The solution was magnetically stirred and placed at 90 °C and 350 mbar. After 4 h, the pressure was decreased to 100 mbar. After another 4 h, the pressure was finally set to 20 mbar and was kept to this value until the end of the reaction.

*From ferulic-based diols (**7a-b**):*

The ferulic-based diol (150 mg) and diethyl adipate were placed in a Schlenck reactor in equimolar proportions. Then, acetophenone (300 wt % vs. the mass of the monomers) and CALB (20 wt % regarding the mass of the monomers) were added. The solution was magnetically stirred and placed at 90 °C and 350 mbar. After 4 h, the pressure was decreased to 250 mbar and was kept to this value until the end of the reaction.

*Recovery of the polyesters:*

The solution was diluted with chloroform (2 mL) and filtered through cotton wool to remove the catalyst. Then, the polymer was precipitated in a cold methanol bath under vigorous stirring. Afterwards, the solution was centrifuged (8000 RCF, 10 min, 4 °C), and the supernatant was eliminated. Finally, the recovered polymer was dried for 24 h in a vacuum oven at 40 °C before analysis.

## 3. Results and Discussion

### 3.1. Polyesters Based on Ferulic Diesters

**Scheme 2:**
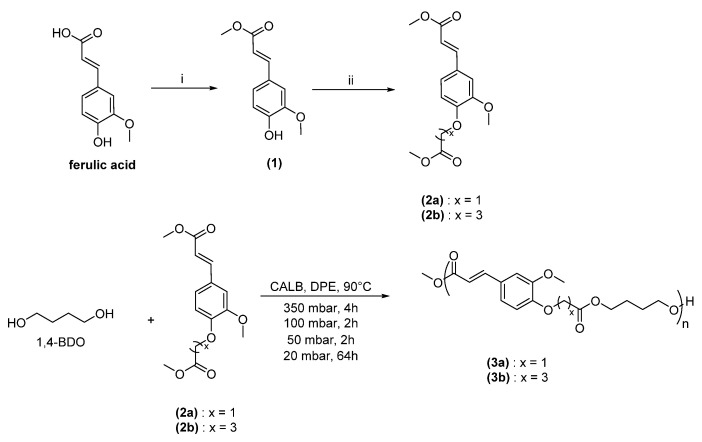
Synthesis and polymerization of the ferulic diesters **2a** and **2b**, i: MeOH, H_2_SO_4_, 70 °C 16 h. ii(a): Methyl chloroacetate, K_2_CO_3_, KI, acetonitrile, 85 °C 16 h. ii(b): Methyl 4-chlorobutyrate, K_2_CO_3_, KI, acetonitrile, 85 °C 16 h.

Various ferulic acid derivatives were synthesized and enzymatically polymerized. The first step has been to modify the ferulic acid to optimize its reactivity toward CALB. Indeed, CALB catalyzed polymerization results in faster kinetics and higher molar masses by transesterification with methyl or ethyl esters than by polycondensation [41,42,43]. The small alcohol adduct in transesterification can easily be eliminated, compared to water, shifting the equilibrium of the reaction. Therefore, ferulic acid was first esterified to give methyl ferulate (**1**) (confirmed by ^1^H and ^13^C NMR, see Supplementary Materials Sections Figures S1 and S8). Ferulic acid is a bifunctional molecule able to undergo homopolymerization through polycondensation [44]. Homopolymers of ferulic acid give incredibly rigid polyesters with high T_g_ [27], making them difficult to process because of their extreme melting temperature and difficulty to dissolve in common solvents. In addition, phenols, such as the one in methyl ferulate, have a lower reactivity compared to primary alcohols, especially in CALB-catalyzed transesterification [24,45]. For all these reasons, ferulic acid is often modified before polymerization [28,29,31]. This allows at the same time to fine-tune the properties of the monomer and those of the resulting polymer, especially of the chain rigidity. Thus, methyl ferulate was modified through Williamson ether synthesis with chloroesters of different lengths (**2a-b**) (Scheme 2). This process allowed to overcome the low reactivity of the methyl ferulate phenol group toward CALB. The structure of the obtained monomers was confirmed by ^1^H and ^13^C NMR (see Supplementary Materials Sections Figures S2, S3, S9 and S10). The resulting products were diesters capable of undergoing enzymatic transesterification with CALB.

**Figure 1:**
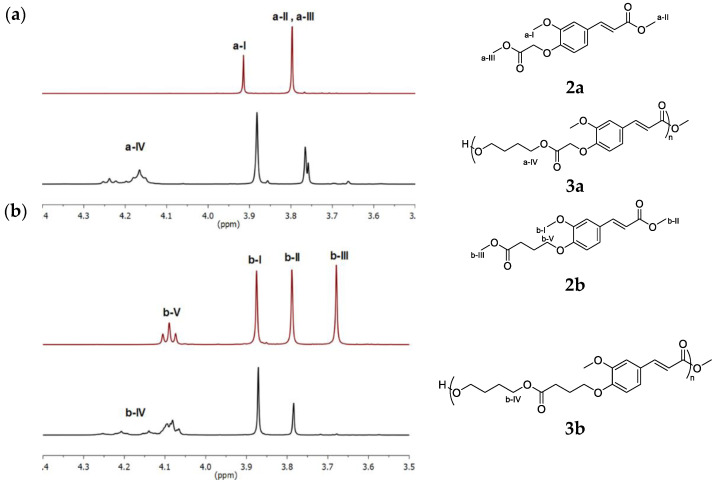
NMR spectra in CDCl_3_ of the ferulic-based diesters and their corresponding polyesters: (**a**) **2a** and **3a**; (**b**) **2b** and **3b**.

The obtained diesters were assayed for CALB-catalyzed polymerization with 1,4-BDO in diphenyl ether under reduced pressure. Then, the obtained products were analyzed by ^1^H NMR (Figure 1 and Supplementary Materials Sections Figures S17 and S18).

The appearance of characteristic peaks (a-IV and b-IV on Figure 1) of the (C**H**_2_-OC(O)) protons from esters between δ = 4.05 and 4.30 ppm confirms the transesterification of the methyl esters with 1,4-BDO. For both monomers **2a** and **2b**, the intensity of the characteristic peaks of the methyl ester functions (respectively a-II, a-III on Figure 1 at δ = 3.72 ppm for **2a** and b-II at δ = 3.79 ppm and b-III at δ = 3.68 ppm on Figure 1 for **2b**) decreased after polymerization when compared with the starting material. This confirms that the methyl ester moieties were consumed during the reaction and therefore converted by CALB. However, the intensities of the end groups signals a-II, a-III, and b-II in the products are still important, indicating a low conversion of the monomer and consequently low molar mass chains. This was confirmed by SEC analysis (see Table 1 and Supplementary Materials Sections Figure S23) that gave molar masses of 1300 g·mol^−1^ and 2000 g·mol^−1^ for **3a** and **3b**, respectively.

The signals corresponding to the two esters of the ferulic diester **2b** can be distinguished and were unambiguously attributed thanks to 2D NMR (Figure 1 and Supplementary Materials Sections Figures S15 and S16). The low intensity of the peak corresponding to the remaining terminal methyl of the methyl butyrate moiety (b-III) in the product suggests a quantitative conversion of this ester function. On the contrary, the intensity of the peak corresponding to the terminal protons of the ester neighboring the double bond (b-II) is only reduced by half after the reaction (Figure 1b and Supplementary Materials Sections Figure S18). This suggests a lower conversion and thus a lower reactivity of this ester function toward CALB. Therefore, this poorly reactive function could rapidly become a terminal group at both ends of the growing chains and thus strongly limit their growth, leading to low molar masses.

The low reactivity of the ferulic acid and its corresponding ester toward CALB has already been described in the literature [46,47,48], showing that *para* and *ortho* hydroxyl and methoxy substituents on the aromatic ring of coumaric derivatives had an inhibitory effect on the CALB activity in esterification and transesterification. Computational modeling performed by Otto et al. [49] suggests that steric hindrance cannot explain this loss of reactivity toward specific substitutions and rather indicates that charge distribution could be the cause. We observe here that this lack of reactivity hindered the synthesis of high molar mass polyesters after 72 h of reaction, resulting in oligoesters with a DP_n_ up to 5.

### 3.2. Polyesters Based on Hydrogenated Ferulic Diesters

The hydrogenation of the double bond has been shown to unlock the reactivity of ferulic acid [47,48]. This method was previously used for the CALB-catalyzed synthesis of ferulic-based monomers [34]. It allowed a high conversion of the substrate in only 4 h. We used a similar strategy to improve the reactivity of the studied monomers toward CALB but this time in a process of enzymatic polymerization.

**Scheme 3:**
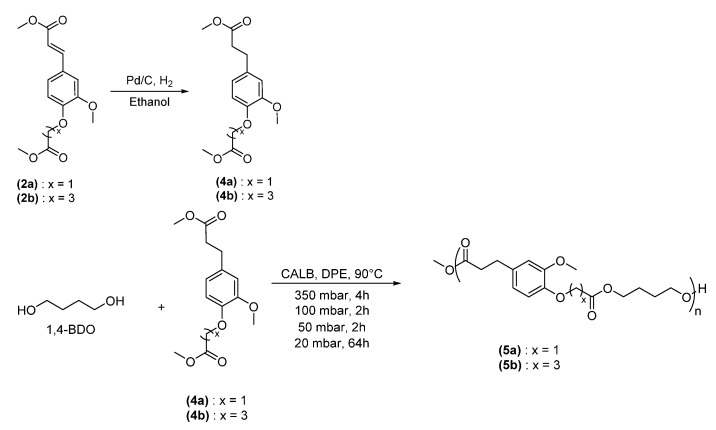
Synthesis and polymerization of the hydrogenated ferulic diesters **4a** and **4b**.

**Figure 2:**
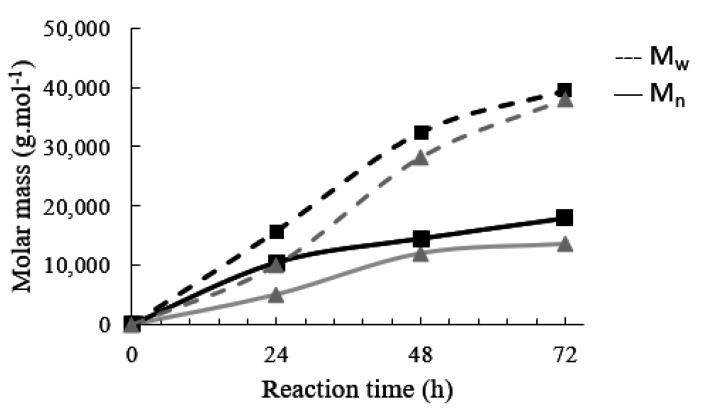
Molar mass of the polymers synthesized from the hydrogenated monomers. 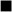 **5a**, 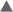 **5b**.

The monomers **2a** and **2b** were hydrogenated using Pd/C as catalyst in ethanol to obtain **5a** and **5b**. The structure of the products was confirmed by ^1^H and ^13^C NMR (see Supplementary Materials Sections Figures S4, S5, S11 and S12). Then, they were polymerized with 1,4-BDO (Scheme 3). The reaction was monitored by SEC analysis of samples withdrawn at regular intervals (Figure 2).

As expected, the reactivity of the ferulic moieties was greatly enhanced by hydrogenation. Quantitative conversion of the monomers **4a** and **4b** was observed from NMR analyses in Supplementary Materials Sections Figures S19 and S20. From NMR analysis of **5a**, it is possible to distinguish the signal corresponding to the C**H**_2_ of the diol linked to the ester vicinal to where the double bond at δ = 4.19 ppm and the C**H**_2_ of the diol linked to the ester coming from the Williamson ether synthesis at δ = 4.06 ppm. Both peaks have similar intensity, indicating that the diol has bound to both esters without distinction. This result would suggest that both esters have an equivalent reactivity toward CALB.

The polyesters **5a** and **5b** were obtained with high M_w_ of 39,400 g·mol^−1^ and 38,000 g·mol^−1^, respectively, as measured by SEC (see Supplementary Materials Sections Figure S24 and Table 1). It is interesting to notice that **5a** systematically resulted in higher DP_n_ than **5b** (58 for **5a** compared to 37 for **5b**). This result is counterintuitive and quite unexpected, since previous studies showed that CALB presented a higher activity toward aliphatic monomers with a number of carbons higher than 4 [9,50,51]. A higher reactivity of **4b** in comparison to **4a** would be expected, as previous studies showed higher reactivity of the ester function with increasing distance from the bulky aromatic ring [49]. However, prediction of the enzyme activity cannot be only based on steric hindrance considerations because of the complex interactions with the substrate [48]. The M_n_ of **5a** (17,900 g·mol^−1^) is comparable to values obtained for equivalent aliphatic polyesters enzymatically synthesized with 1,6-HDO. However, the aromatic polyesters show lower DP_n_ compared to aliphatic ones because of the high molar mass of the ferulic-based diesters (58 for **4a** compared to 81–83 in the literature [52]).

Thus, the activity of CALB toward the ferulic-based diester was efficiently unlocked by hydrogenation of the double bond, leading to high molar mass polyesters. As depicted before, two main hypotheses could explain this result. First, the hydrogenation of the double bond lowers the rigidity of the molecule, facilitating its introduction and orientation in the active site of the enzyme. The second hypothesis is that the electronic conjugation between the ester and the aromatic ring is broken by the disappearance of the double bond. This conjugation could lower the reactivity of the ferulic esters [42,47,49]. Indeed, because of its double bond, ferulic acid presents multiple resonance forms, making it less prompt to nucleophilic attack from the serine amino acid of the CALB active site, hindering enzymatic activity.

**Figure 3:**
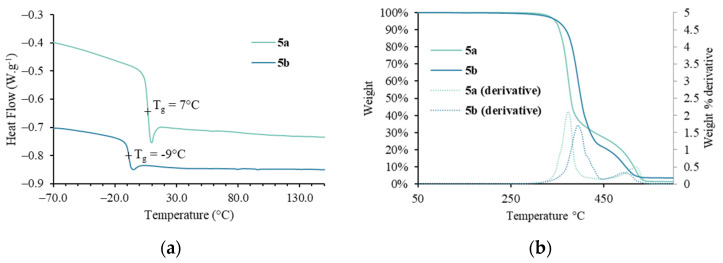
Thermal analysis of **5a** and **5b**: (**a**) DSC measurement, heating rate 10 °C/min, data have been offset one from another by −0.5 W·g^−1^; (**b**) TGA measurement under air at a heating rate of 10 °C/min.

The thermal properties of the obtained polyesters were assessed (Figure 3 and Table 1). Both polyesters **5a** and **5b** were fully amorphous with a T_g_ measured by DSC (Figure 3a) at 7 and −9 °C, respectively. It is the first time these monomers are used for polymer synthesis and no similar structures, even synthesized by any other means than enzymatic catalysis, could be found in the literature for comparison. Polyesters **5a** and **5b** present low T_g_ in comparison to fully ferulic-based polyesters showing a T_g_ above 113 °C [27]. The hydrogenation of the monomer’s double bond as well as the use of an aliphatic diol leads to a polymer structure with a lower T_g_. However, when compared to fully aliphatic polyesters synthesized from 1,4-BDO such as polybutylene adipate (PBAd) or polybutylene succinate (PBS) (with a T_g_ of −59 and −37 °C, respectively [9]), the use of the ferulic-based hydrogenated diester increased the T_g_ of the polyesters. These results are in agreement with previous studies showing that adding ferulic-based units to a polymeric structure leads to mostly amorphous materials with increased T_g_ [38,53]. Finally, the polyester synthesized from methyl 4-chlorobutyrate-based diester (**5b**) presents a lower T_g_ compared to the one based on methyl chloroacetate (**5a**). Indeed, its longer aliphatic moiety induces more chain flexibility, reducing the T_g_ of the final polymer.

The polymers were also analyzed by TGA, and the weight loss curves are presented in Figure 3b. The temperature of degradation at the maximum rate (T_dmax_) was measured at 373 °C and 394 °C for **5a** and **5b**, respectively. These values are within the range of those found for aliphatic polyesters such as PBAd and PBS (391 and 355 °C, respectively [9]). Interestingly, the degradation of both polyesters **5a** and **5b** occurred in two steps. This degradation pattern has already been observed in other aromatic polyesters such as PET [54]. The first step could correspond to the thermal degradation of the aliphatic parts of the polymer and correspond to the temperature of the ester bonds breaking. Then, the aromatic parts get reorganized into more thermally stable aromatic polycyclic ashes, which get decomposed during the second step occurring at a much higher temperature.

### 3.3. Polyesters Based on Ferulic Diols

**Scheme 4:**
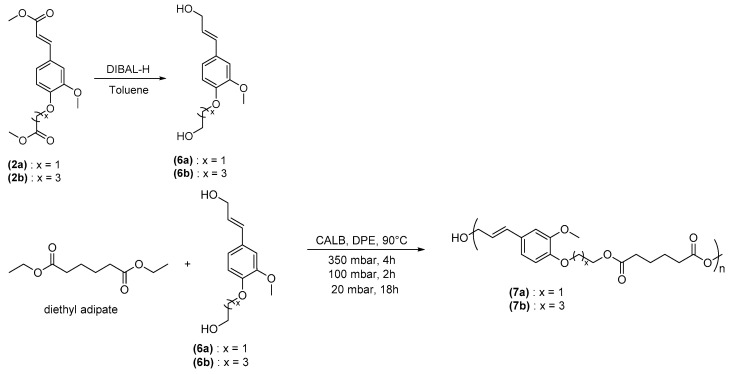
Synthesis and polymerization of the ferulic-based diols **6a** and **6b**.

Another strategy of ferulic acid modification was evaluated. Reducing the methyl ferulate to its corresponding alcohol could offer a similar effect to hydrogenation. Indeed, after the reduction, the monomer gains a CH_2_ and thus flexibility (Scheme 4), which could favor the accessibility of the molecule to the enzyme active site. Moreover, the alcohol resulting from the reduction would not be electronically conjugated to the aromatic ring. Additionally, along this synthesis path, the monomer double bond is preserved, bringing rigidity to the chain backbone and allowing further modification of the polymer [44,55].

Both ferulic-based diesters previously synthesized (**2a** and **2b**) have been reduced to their corresponding diols by diisobutyl aluminum hydride (DIBAL-H). The structure of the monomers was confirmed by ^1^H and ^13^C NMR (see Supplementary Materials Sections Figures S6, S7, S13 and S14). Then, the monomers were evaluated for the synthesis of polyesters via CALB-catalyzed polymerization, as presented in Scheme 4. Diethyl adipate has been selected as a co-monomer, since previous studies demonstrated its good reactivity with CALB, thus maximizing the chances of obtaining a high molar mass polyester [50,56].

**Figure 4:**
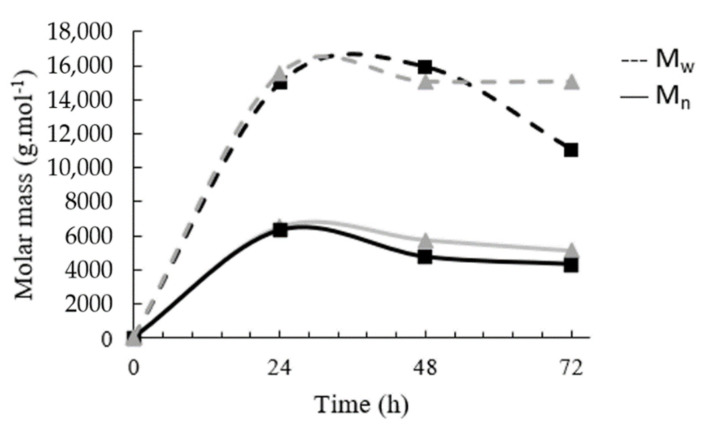
Molar masses of the polyesters synthesized from the ferulic-based diols. 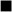 **7a**, 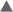 **7b**.

The obtained polyester **7a** and **7b** were analyzed by NMR (Supplementary Materials Sections Figures S21 and S22) and SEC (Supplementary Materials Sections Figure S25). The M_n_ of **7a** and **7b** determined by SEC after 24 h were respectively of 6500 and 6300 g·mol^−1^ corresponding to a DP_n_ of 19 and 17. The two polyesters were obtained with similar molar masses and kinetics, which is in agreement with the study by Pellis et al. [57], who showed that moving the hydroxyl group further away from the aromatic ring did not significantly alter the molar mass of the obtained polyesters. Longer times of polymerization did not increase the polymers’ molar masses. On the contrary, a decrease in M_n_ was even observed after 24 h of reaction (Figure 4). Side reactions could be responsible for such a decrease in molar masses. To investigate this phenomenon and to understand more deeply the structure of the formed polymeric chains, the synthesized polyesters have been analyzed by MALDI-TOF MS.

**Figure 5:**
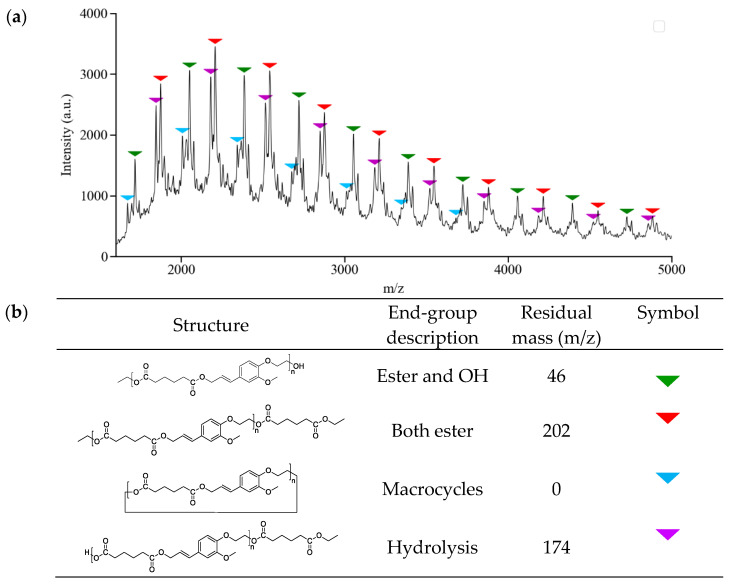
(**a**) MALDI-TOF MS spectrum of **7a**; (**b**) corresponding identified polymer structures.

Although the large polydispersity of the polymer does not allow obtaining quantitative results from the MALDI-TOF MS [58], it is possible to qualitatively compare the end groups with each other. A large number of polymeric chains are ended with both an ester and a hydroxyl group (green triangles in Figure 5a). This is expected due to the one-to-one stoichiometry of the monomers. However, a significant proportion of polymeric chains are terminated on both ends by an ester group (red triangles in Figure 5a). This result indicates an unbalanced stoichiometry in the reaction medium with an excess of the diester. Side reactions involving one of the monomers could result in its deactivation or loss of reactivity and thus unbalance the stoichiometry of the reaction. However, no obvious traces of side products could be found on the NMR analyses. Moreover, reacting the diol alone with and without CALB in the presence of ethanol did not allow clearly identifying any side product from **6a**. Using an excess of diol resulted in polymers with a majority of chains terminated with both an ester and a hydroxyl group (Supplementary Materials Sections Figure S26 and Table S1). However, the polymer presented similar or lower molar masses (Supplementary Materials Sections Figure S27 and Table S2) than for the synthesis with a stoichiometric ratio. The end group analysis also showed traces of hydrolysis with chains ended with both an acid and an ester, resulting in a residue m/z equal to 174 (purple triangles in Figure 5a). This phenomenon could also explain the end of chain growth followed by the slight decrease in molar masses (Figure 4). Finally, macrocycles were detected, and although they were present in a lower amount compared to other end groups, they could also contribute to lowering the apparent molar mass determined by SEC. 

**Figure 6:**
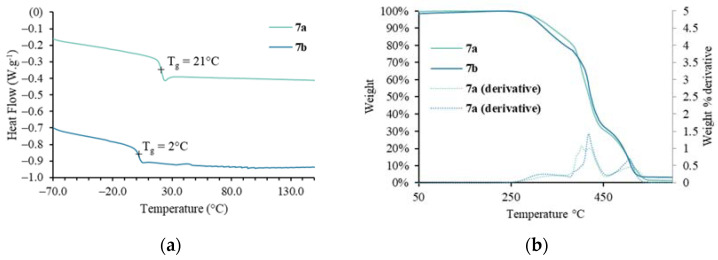
Thermal analysis of **7a** and **7b**: (**a**) DSC measurement, heating rate 10 °C/min, data have been offset one from another by −0.5 W·g^−1^; (**b**) TGA measurement, under air at a heating rate of 10 °C/min.

The thermal properties of the polyesters have been assessed by DSC and TGA (Figure 6 and Table 1). The Tg of **7a** and **7b** were measured at 21 °C and 2 °C, respectively. Once again, the materials synthesized are fully amorphous and therefore present no melting or crystallization temperatures. However, despite a lower molar mass, polyester **7a** presents the highest Tg when compared with all other polymers from this study. As expected, the preservation of the double bond resulted in a more rigid polymeric backbone and therefore a higher Tg. This observation is in agreement with recent results from Prezzana et al. [59]. The diol originating from methyl 4-chlorobutyrate (**6b**) gives a polyester with lower Tg compared to the diol obtained from methyl chloroacetate (**6a**). Again, the longer aliphatic chain of monomer 6b decreases the Tg of the resulting polyester **7b**. The Tg of this polyester was even lower than the Tg measured for polyesters synthesized from the methyl 4-chlorobutyrate hydrogenated-based diester (**5b**). However, these results are not fully com-parable, since in 7b, the aromaticity is brought by the diol when in **5b**, it comes from the diester. Thus, these polyesters present aliphatic co-monomers (1,4-BDO and DEA) that are not directly comparable and could have an impact on the thermal transitions of the final material.

The thermal degradation of **7a** and **7b** was measured by TGA. A similar degradation behavior was observed for both polymers. When compared with **5a** and **5b**, the degradation begins at lower temperatures with a T_d5%_ of only 316 and 308 °C for **7a** and **7b**, respectively. This early degradation could be due to the presence of the double bond of the α,β-unsaturated alcohol (coniferyl). It is known to form by-products such as aldehydes through radical pathways during thermal degradation [60]. The degradation of the polymers appears as three steps with maximum rates of degradation at 343, 403, and 506 °C for **7a** and 321, 418, and 507 °C for **7b**. As depicted before, the first step of degradation could correspond to the degradation linked to the presence of coniferyl alcohol moieties. The second degradation step is the fastest and is in the temperature range of degradation of aliphatic polyesters. The final degradation step corresponds to the aromatic part of the polyesters that only get fully degraded above 530 °C, as observed for the previous polymers.

## 4. Conclusions

In this study, several various monomers derived from ferulic acid were synthesized and tested for CALB-catalyzed polymerization. First, ferulic acid was converted into a long and a short diester. With both substrates, only partial monomer conversion by CALB was observed, resulting in low molar mass oligomers. This observation was in good agreement with studies in the literature pointing to the α,β-unsaturation as responsible for the lack of reactivity of ferulic compounds. Thus, α,β-unsaturation of the monomers was eliminated by hydrogenation prior to their assessment for CALB-catalyzed polymerization. The hydrogenation of the monomers allowed the synthesis of polyesters with the highest molar masses in this study, with M_w_ up to 33,100 g·mol^−1^ for the shorter diester (**4a**). The M_n_ of the polyester (17,900 g·mol^−1^) is comparable to values obtained for equivalent fully aliphatic polyesters synthesized by CALB-catalyzed polymerization. However, lower DP_n_ (58 for **4a**) values were obtained with aromatic monomers when compared to aliphatic ones (81–83 [52]) due to the high molar mass of the ferulic-based diester monomer. Moreover, hydrogenation of the ferulic-based monomers led to polymer T_g_ up to 7 °C, which was below ambient temperature. Another strategy used in this study was the reduction of the ferulic diesters into their corresponding diols. The polyesters synthesized from the reduced monomers resulted in M_w_ up to 15,500 g·mol^−1^. However, the presence of side reactions (hydrolysis, cyclization) was observed in the medium, explaining the lower molar mass obtained when compared to the hydrogenated monomers. However, in line with existing literature, it has been shown that preserving the monomer double bond, as in the reduction of the ferulic diesters, stiffens the polymer chain backbone, resulting in higher T_g_ (up to 21 °C). However, although the presence of the double bond increases the T_g_ of the polymer, it could constitute its weak point during thermal degradation, resulting in an early thermal degradation when compared with the other polyesters.

This study constitutes a first step in understanding the optimal condition for the CALB-catalyzed synthesis of ferulic-based aromatic polyesters. Other modifications of ferulic acid as well as the combination of the previously synthesized monomers with other co-monomers will be investigated. Indeed, such monomers could give access to biobased polymers with innovative properties that could challenge conventional petroleum-based materials.

Moreover, the enzymatic degradation of the polymers produced in this study could be investigated in order to reduce their end-of-life environmental impact through recycling [61,62]. Indeed, several studies focused on the CALB-catalyzed depolymerization of polyesters [63,64]. This could allow both the synthesis and recycling of ferulic-based polyester using the same enzyme.

## Figures and Tables

**Table 1:** Main properties of the enzymatically synthesized polyesters.

Ferulic-Based Monomer	Co-Monomer	Yield ^a^	M_n_ (g·mol^−1^)	M_w_ (g·mol^−1^)	DP_n_	T_g_ (°C)	T_dmax_ (°C)	T_d5%_ (°C)
(**2a**) 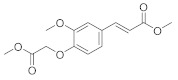	1,4-BDO	n.d. ^b^	1300	1600	4	n.d. ^b^	n.d. ^b^	n.d. ^b^
(**2b**) 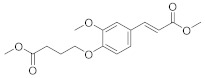	1,4-BDO	n.d. ^b^	2000	2500	5	n.d. ^b^	n.d. ^b^	n.d. ^b^
(**4a**) 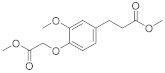	1,4-BDO	58%	17,900	33,100	58	7	373	346
(**4b**) 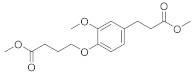	1,4-BDO	96%	12,600	29,800	37	−9	394	350
(**6a**) 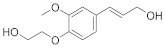	DEA	82%	6500	15,000	19	21	403	316
(**6b**) 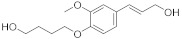	DEA	79%	6300	15,500	17	2	418	308

^a^ Recovered mass yield taking into account the loss of methanol or ethanol during the reaction. ^b^ Not determined as only low molar mass oligomers were obtained.

## Data Availability

Not applicable.

## Supplementary Materials

The following are available online at https://www.mdpi.com/article/10.3390/polym13213693/s1, **Figure S1.** ^1^H NMR analysis of methyl ferulate; **Figure S2.** ^1^H NMR analysis of methyl 4-(methyl ethanoate-oxy)-ferulate (**2a**); **Figure S3.** ^1^H NMR analysis of methyl 4-(methyl butanoate-oxy)-ferulate (**2b**); **Figure S4.** ^1^H NMR analysis of methyl 3-(3-methoxy-4-(2-methoxy-2-oxoethoxy)phenyl)propanoate (**4a**); **Figure S5.** ^1^H NMR analysis of methyl 4-(2-methoxy-4-(3-methoxy-3-oxopropyl)phenoxy)butanoate (**4b**); **Figure S6.** ^1^H NMR analysis of 4-(hydroxyethoxy)-coniferyl alcohol (**6a**); **Figure S7.** ^1^H NMR analysis of 4-(hydroxybutoxy)-coniferyl alcohol (**6b**); **Figure S8.** ^13^C NMR analysis of methyl ferulate (**1**); **Figure S9.** ^13^C NMR analysis of methyl 4-(methyl ethanoate-oxy)-ferulate (**2a**); **Figure S10.** ^13^C NMR analysis of methyl 4-(methyl butanoate-oxy)-ferulate (**2b**); **Figure S11.** ^13^C NMR analysis of methyl 3-(3-methoxy-4-(2-methoxy-2-oxoethoxy)phenyl)propanoate (**4a**); **Figure S12.** ^13^C NMR analysis of methyl 4-(2-methoxy-4-(3-methoxy-3-oxopropyl)phenoxy)butanoate (**4b**); **Figure S13.** ^13^C NMR analysis of 4-(hydroxyethoxy)-coniferyl alcohol (**6a**); **Figure S14.** ^13^C NMR analysis of 4-(hydroxybutoxy)-coniferyl alcohol (**6b**); **Figure S15.** HMBC NMR analysis of methyl 4-(methyl butanoate-oxy)-ferulate (**2b**) in CDCl_3_; **Figure S16.** HSQC NMR analysis of methyl 4-(methyl butanoate-oxy)-ferulate (**2b**) in CDCl_3_; **Figure S17.** ^1^H NMR analysis of poly(butylene-co-4-(methyl ethanoate-oxy)-ferulate) (**3a**); **Figure S18.** ^1^H NMR analysis of poly(butylene-co-4-(methyl butanoate-oxy)-ferulate) (**3b**); **Figure S19.** ^1^H NMR analysis of poly(butylene-co-3-(3-methoxy-4-(2-methoxy-2-oxoethoxy)phenyl)propanoate) (**5a**); **Figure S20.** ^1^H NMR analysis of poly(butylene-co-4-(2-methoxy-4-(3-methoxy-3-oxopropyl)phenoxy)butanoate) (**5b**); **Figure S21.** ^1^H NMR analysis of poly(4-(hydroxyethoxy)-coniferylene -co-adipate) (**7a**); **Figure S22.** ^1^H NMR analysis of poly(4-(hydroxybutoxy)-coniferylene-co-adipate) (**7b**); **Figure S23.** SEC analyses in THF of the ferulic diester-based polyesters **3a** and **3b** (curves were normalised by their area); **Figure S24.** SEC analyses in THF of the hydrogenated ferulic diester-based polyesters **5a** and **5b** (curves were normalised by their area); **Figure S25.** SEC analyses in THF of ferulic diol-based polyesters **7a** and **7b** (curves were normalised by their area); **Figure S26.** MALDI-TOF MS analysis of 7a obtained with 1.1 equivalent of diol in comparison to the diester after 24 h of synthesis in acetophenone at 90°C and under reduced pressure; **Table S1.** Mass of the corresponding end-groups in MALDI-TOF MS analysis of **7a** obtained with 1.1 equivalent of diol in comparison to the diester after 24 h of synthesis in acetophenone at 90°C and under reduced pressure; **Figure S27.** SEC analyses in THF of ferulic diol-based polyesters **7a** synthesized with and without an excess of diol (curves were normalised by their area); **Table S2.** Average molar mass and dispersity from the SEC analyses in THF of ferulic diol-based polyesters **7a** synthesized with and without an excess of diol.
