# Supplementary material for: Ferulic Acid as Building Block for the Lipase-Catalyzed Synthesis of Biobased Aromatic Polyesters
Source: Polymers (Basel). 2021 Oct 27;13(21):3693. doi: 10.3390/polym13213693 (PMC8588094; doi:10.3390/polym13213693)
Supplement: Supplementary file 1 [file polymers-13-03693-s001.zip › polymers-1423824-SI.pdf]

polymers

MDPI

Supporting Information

Ferulic Acid as Building Block for the Lipase-Catalyzed Synthesis of Biobased Aromatic Polyesters

Alfred Bazin, Luc Avérous and Eric Pollet *

BioTeam/ICPEES-ECPM, UMR CNRS 7515, Université de Strasbourg, 25 rue Becquerel, 67087 Strasbourg Cedex 2, France

* Correspondence: eric.pollet@unistra.fr; Tel.: +33(0)3 68 852 786

1. ^{1}H NMR analyses of the monomers

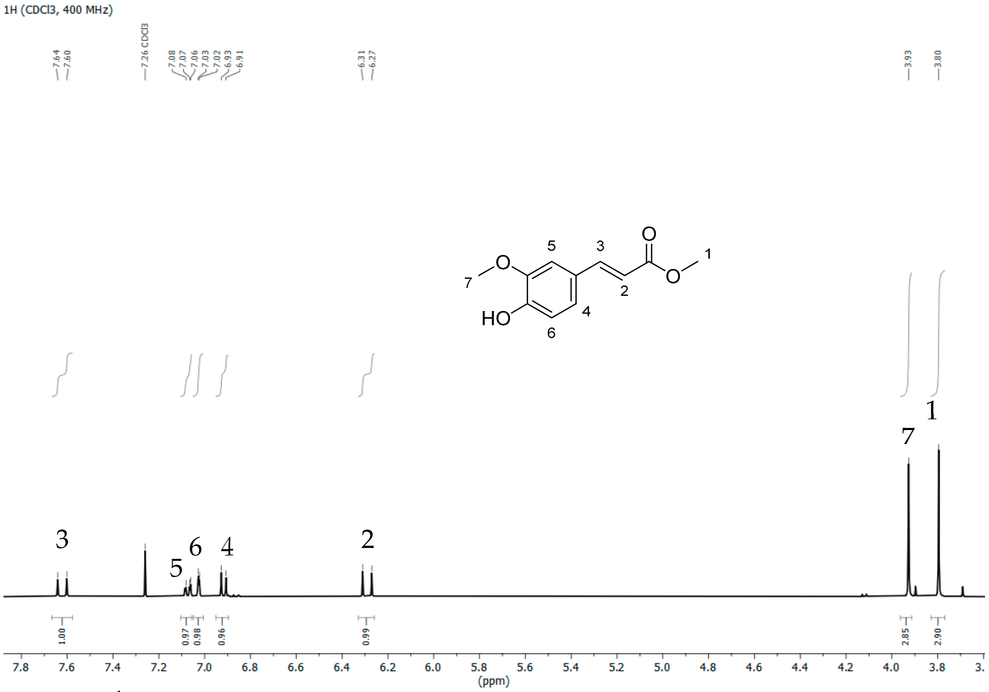

Figure S1. ^{1}H NMR analysis of methyl ferulate (**1**).

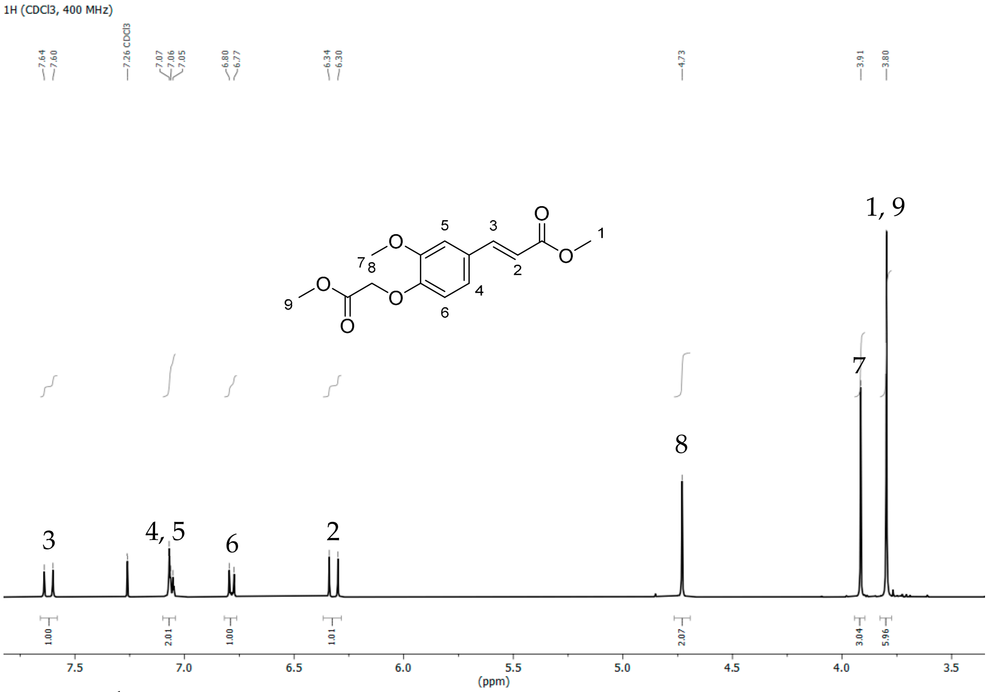

Figure S2. ^{1}H NMR analysis of methyl 4-(methyl ethanoate-oxy)-ferulate (**2a**).

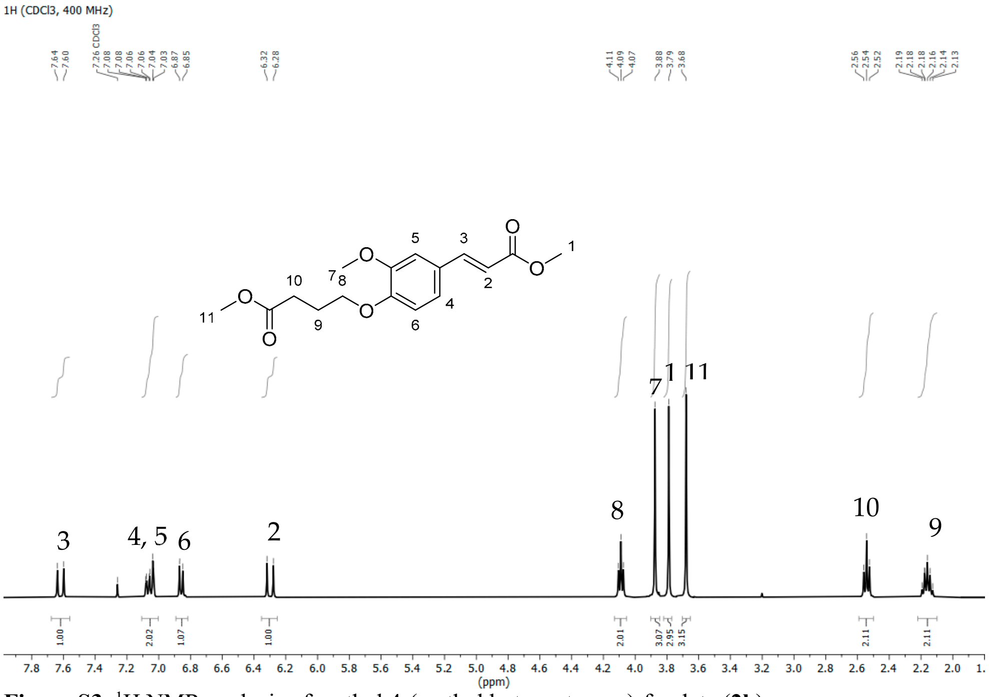

Figure S3. ^{1}H NMR analysis of methyl 4-(methyl butanoate-oxy)-ferulate (**2b**).

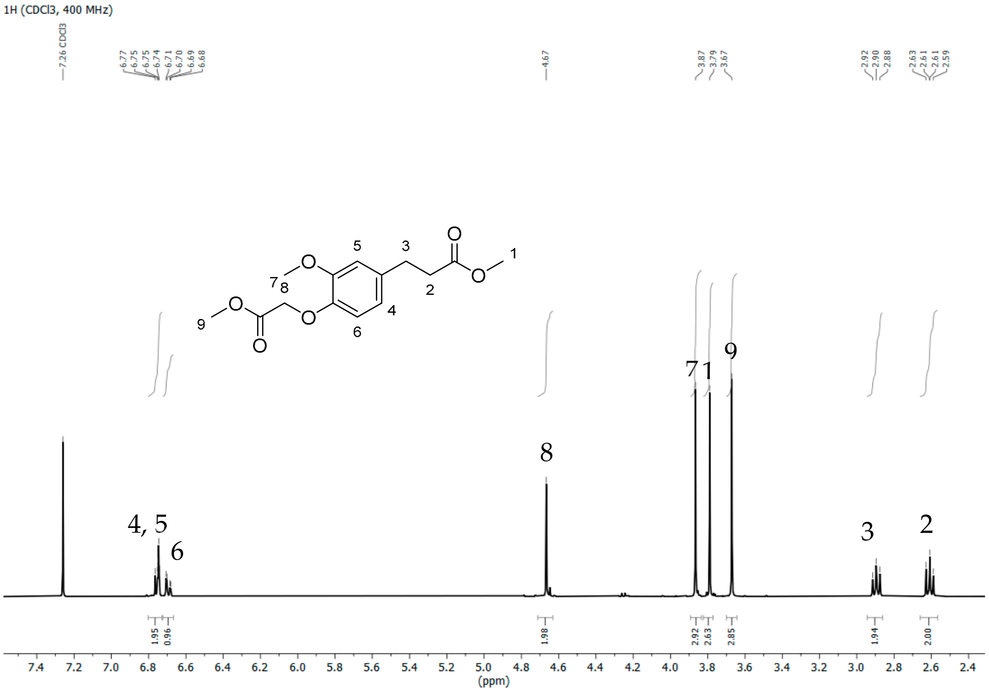

Figure S4. ^{1}H NMR analysis of methyl 3-(3-methoxy-4-(2-methoxy-2-oxoethoxy)phenyl)propanoate (**4a**).

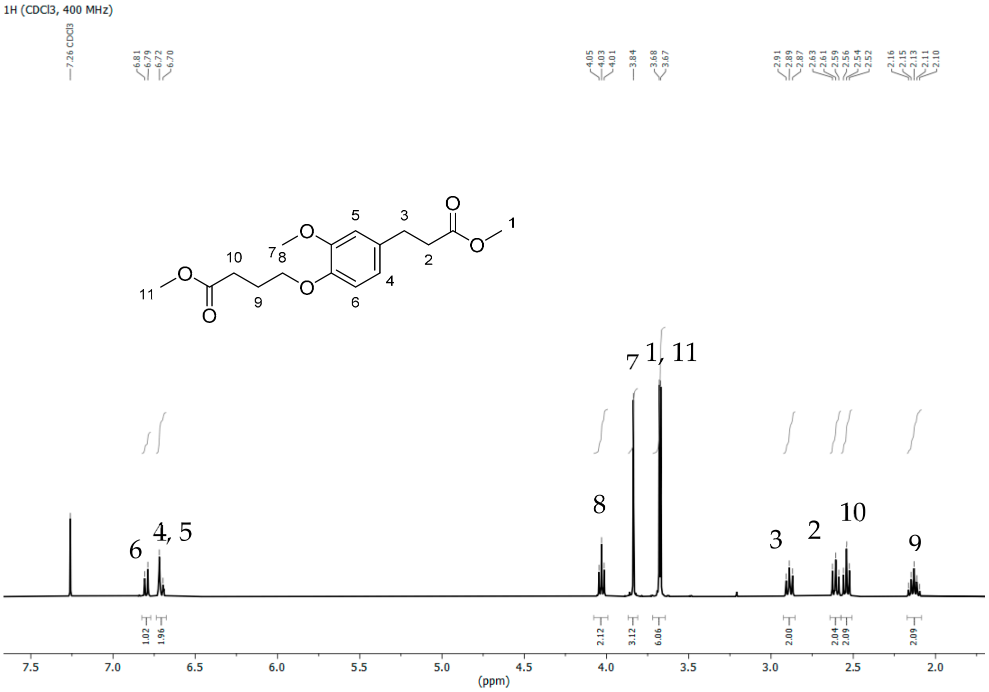

Figure S5. ^{1}H NMR analysis of methyl 4-(2-methoxy-4-(3-methoxy-3-oxopropyl)phenoxy)butanoate (**4b**).

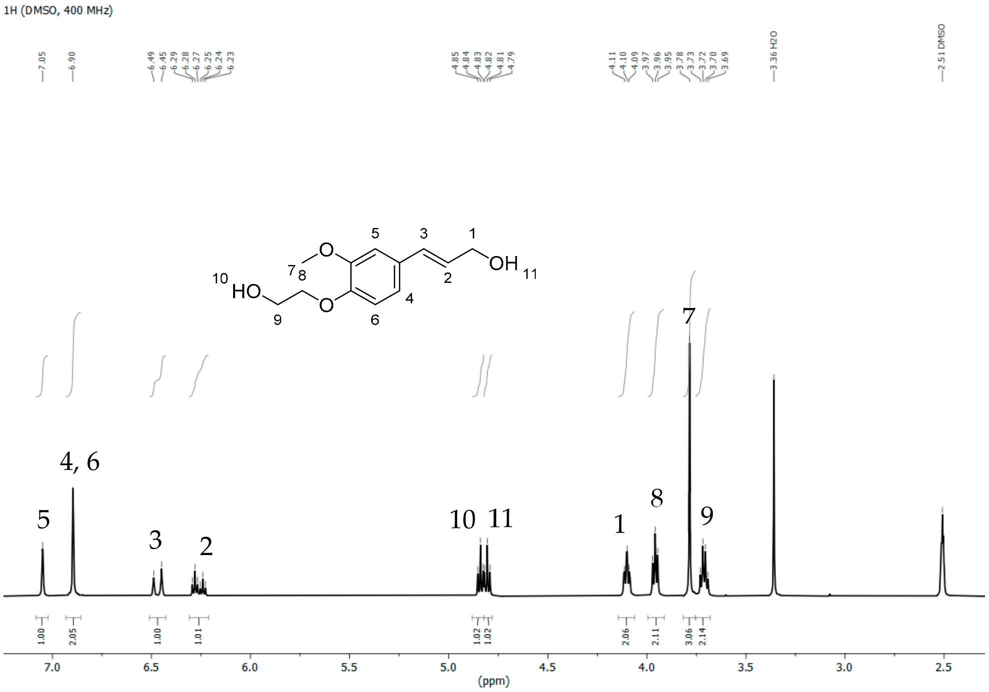

Figure S6. ^{1}H NMR analysis of 4-(hydroxyethoxy)-coniferyl alcohol (**6a**).

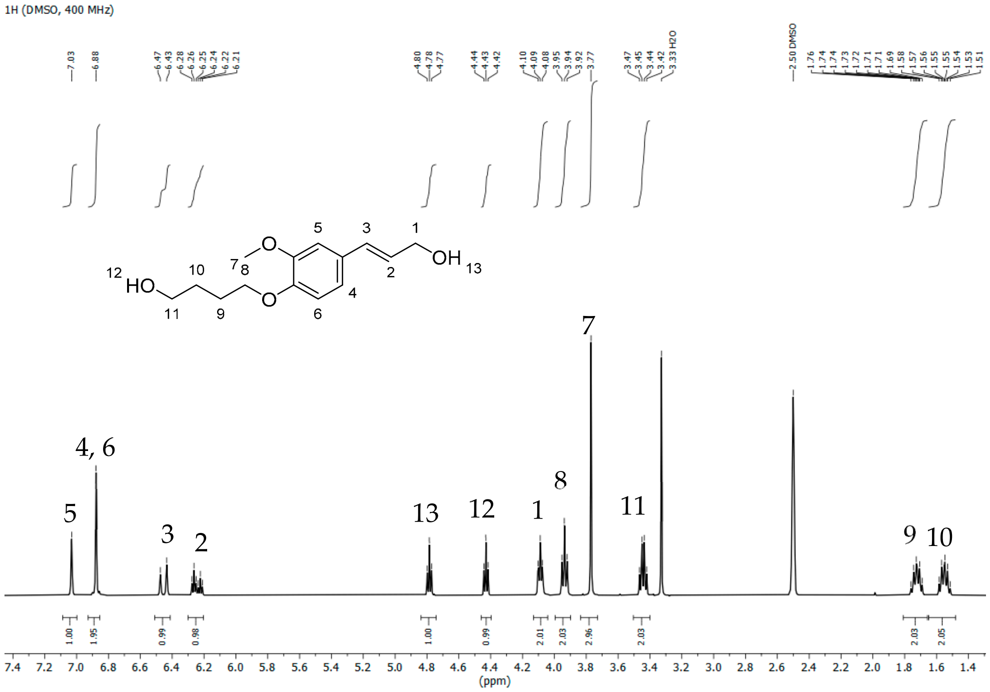

Figure S7. ^{1}H NMR analysis of 4-(hydroxybutoxy)-coniferyl alcohol (**6b**).

2. ^{13}C NMR analysis of the monomers

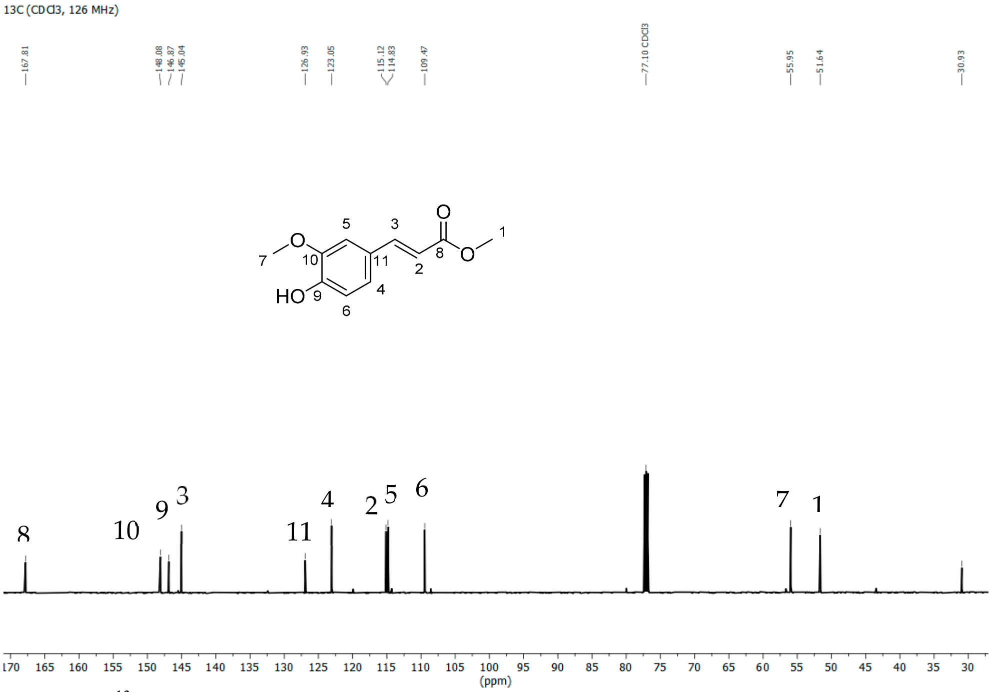

Figure S8. ^{13}C NMR analysis of methyl ferulate (**1**).

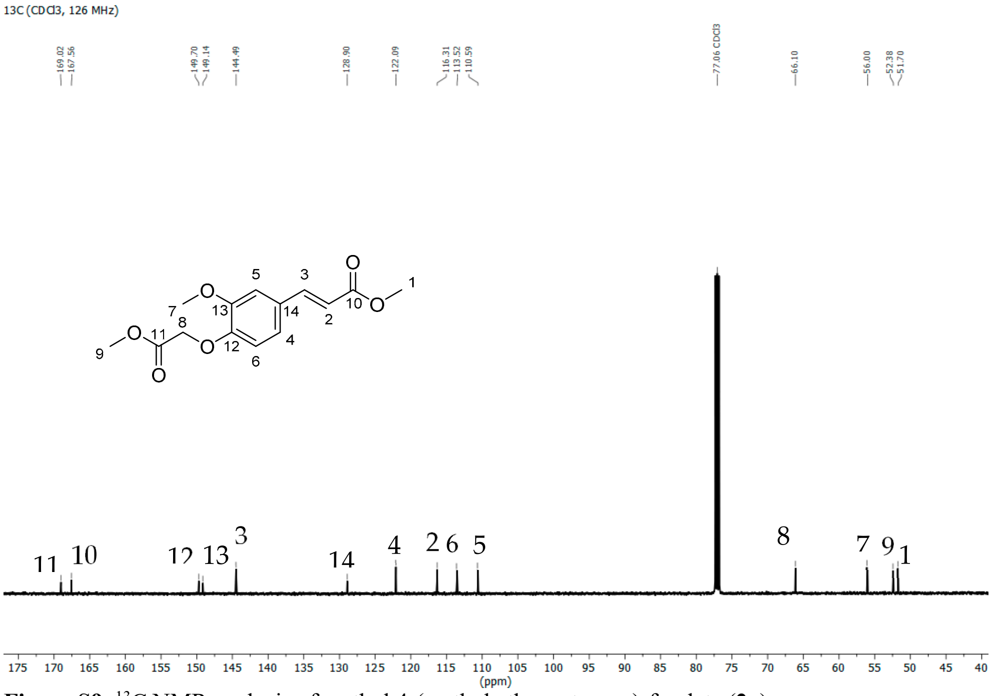

Figure S9. ^{13}C NMR analysis of methyl 4-(methyl ethanoate-oxy)-ferulate (**2a**).

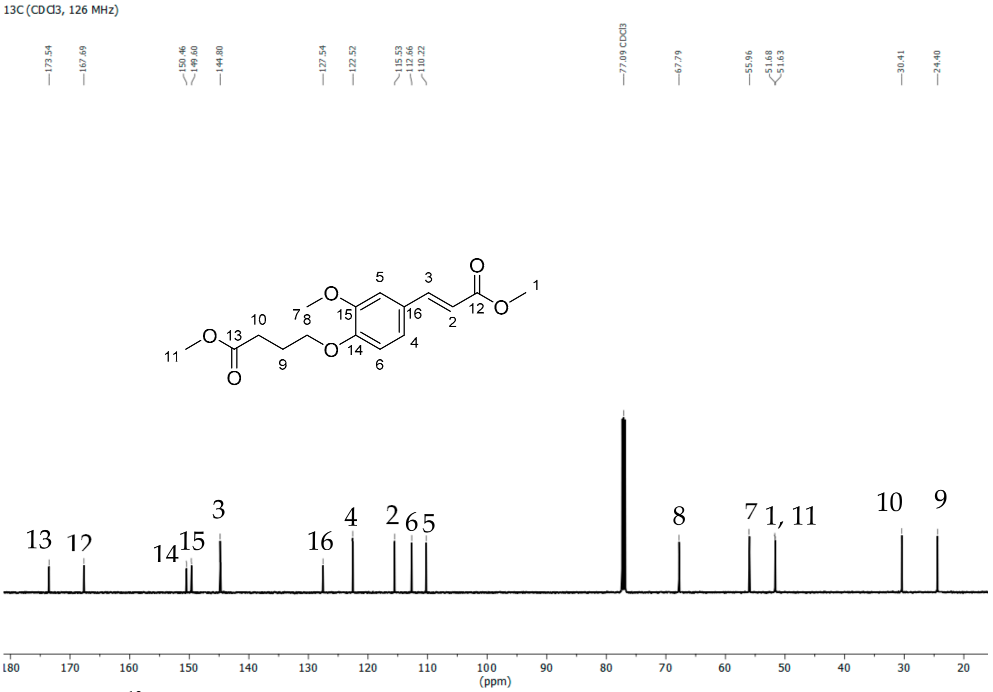

Figure S10. ^{13}C NMR analysis of methyl 4-(methyl butanoate-oxy)-ferulate (**2b**).

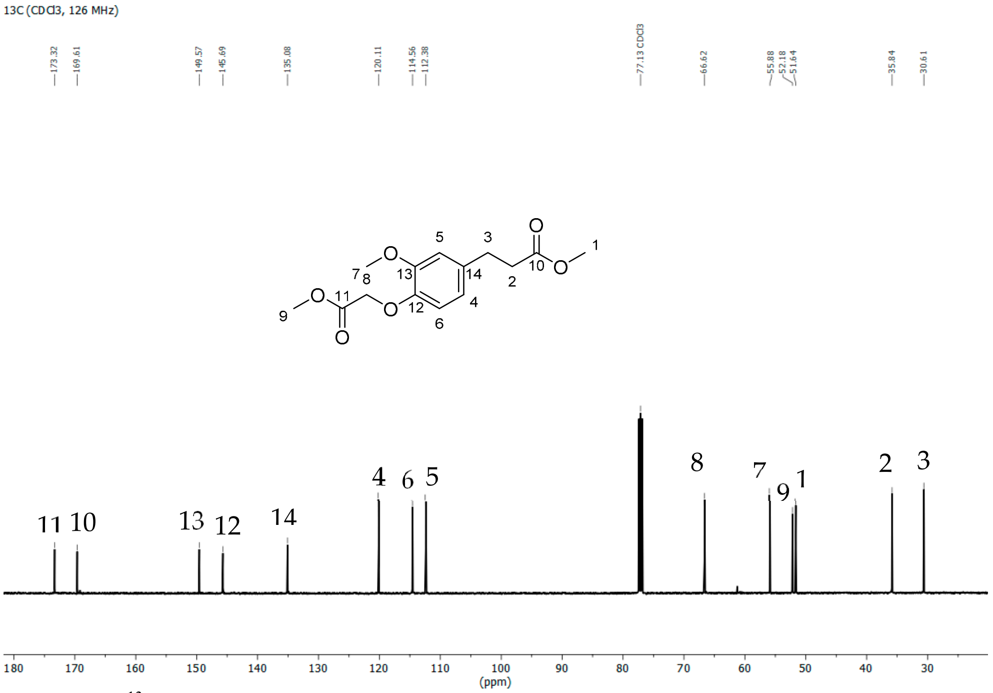

Figure S11. ^{13}C NMR analysis of methyl 3-(3-methoxy-4-(2-methoxy-2-oxoethoxy)phenyl)propanoate (**4a**).

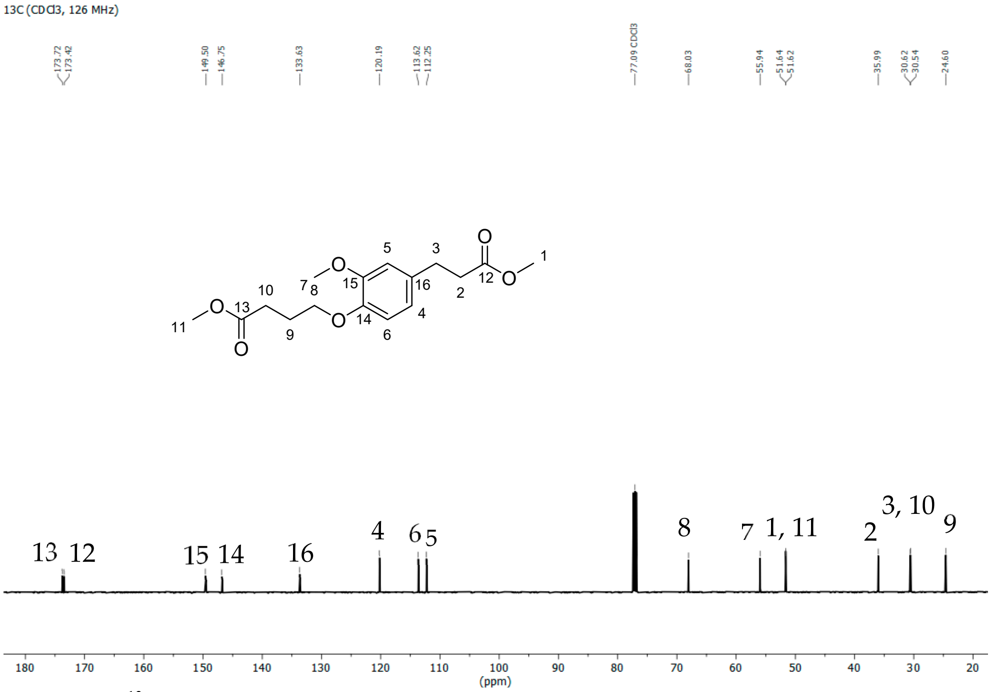

Figure S12. ^{13}C NMR analysis of methyl 4-(2-methoxy-4-(3-methoxy-3-oxopropyl)phenoxy)butanoate (**4b**).

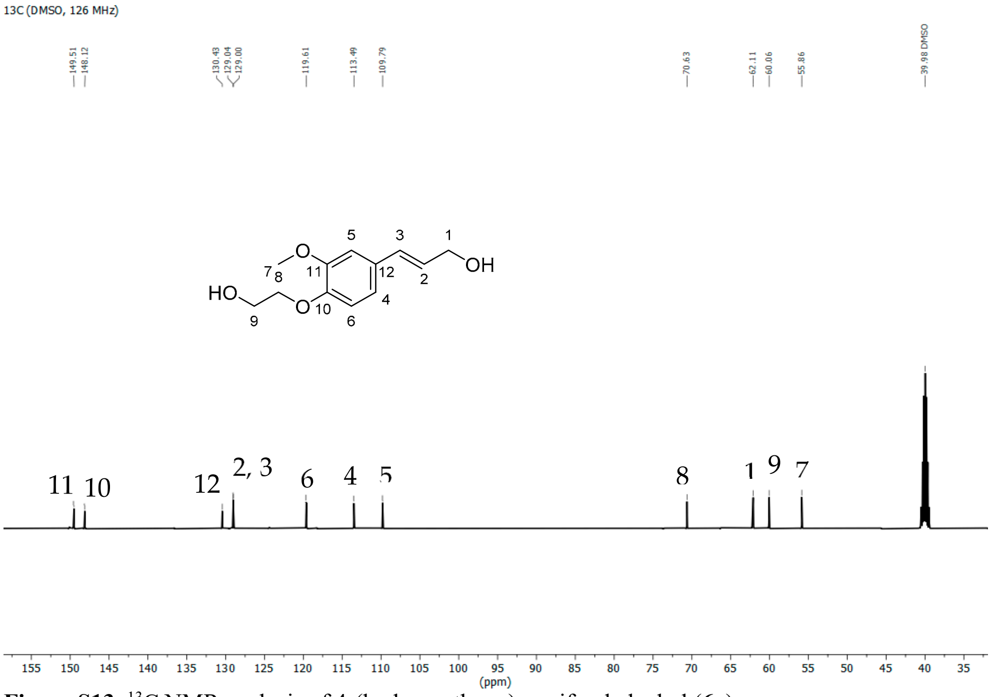

Figure S13. ^{13}C NMR analysis of 4-(hydroxyethoxy)-coniferyl alcohol (**6a**).

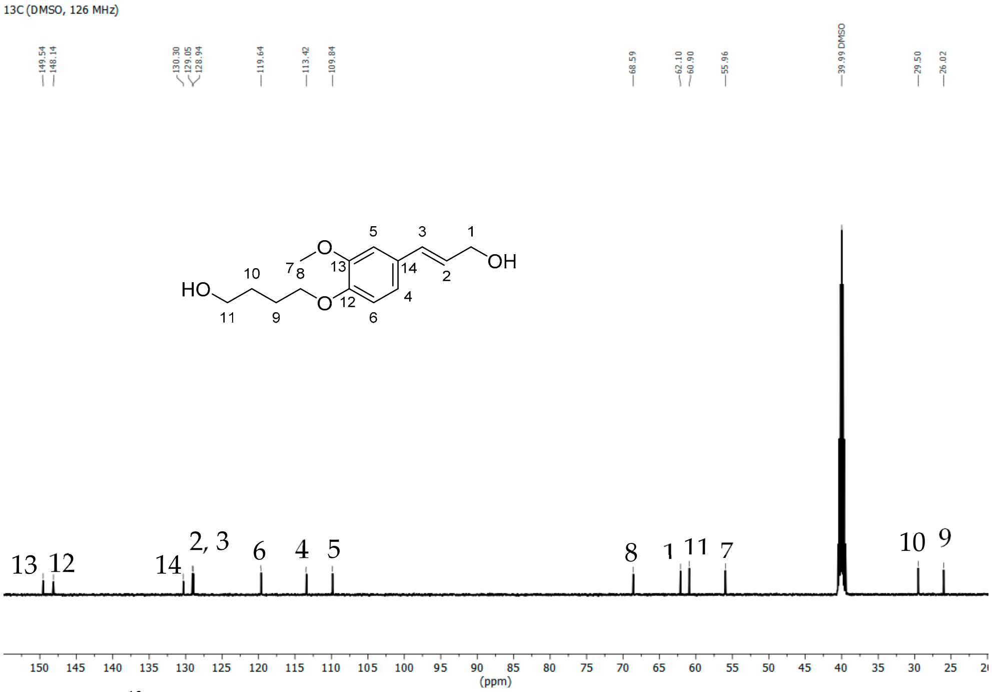

Figure S14. ^{13}C NMR analysis of 4-(hydroxybutoxy)-coniferyl alcohol (**6b**).

3. 2D NMR analyses of 4-(methyl butanoate-oxy)-ferulate (**2b**)

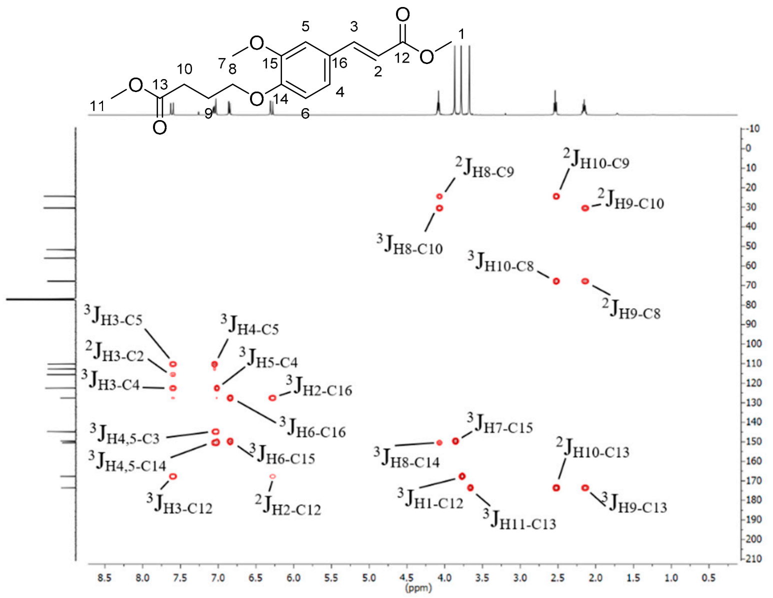

Figure S15. HMBC NMR analysis of methyl 4-(methyl butanoate-oxy)-ferulate (**2b**) in $CDCl_3$.

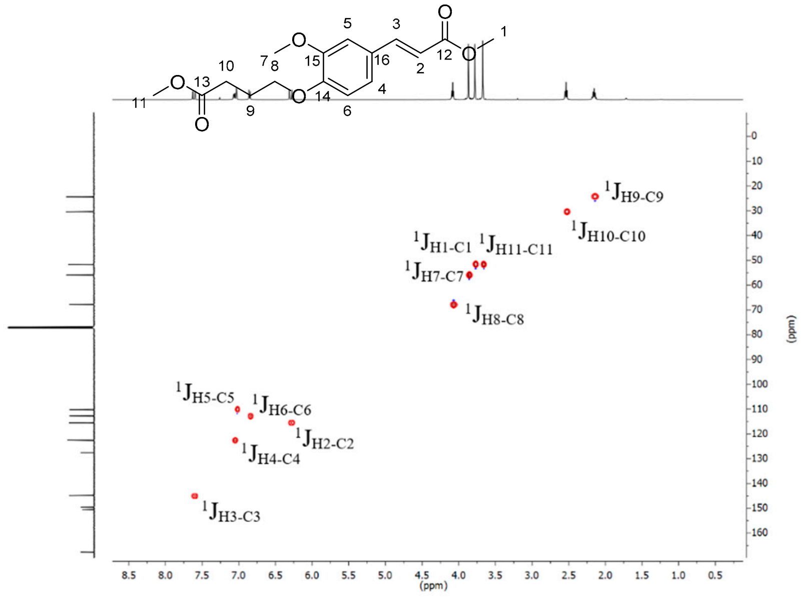

Figure S16. HSQC NMR analysis of methyl 4-(methyl butanoate-oxy)-ferulate (**2b**) in $CDCl_3$.

4. Polymers ^{1}H NMR

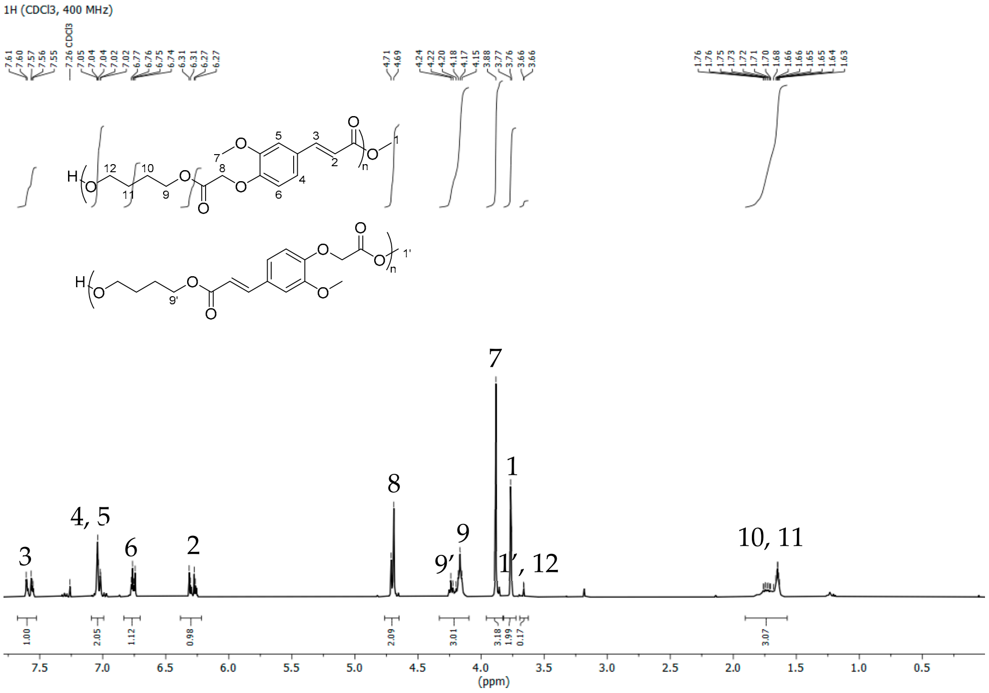

Figure S17. ^{1}H NMR analysis of poly(butylene-co-4-(methyl ethanoate-oxy)-ferulate) (**3a**).

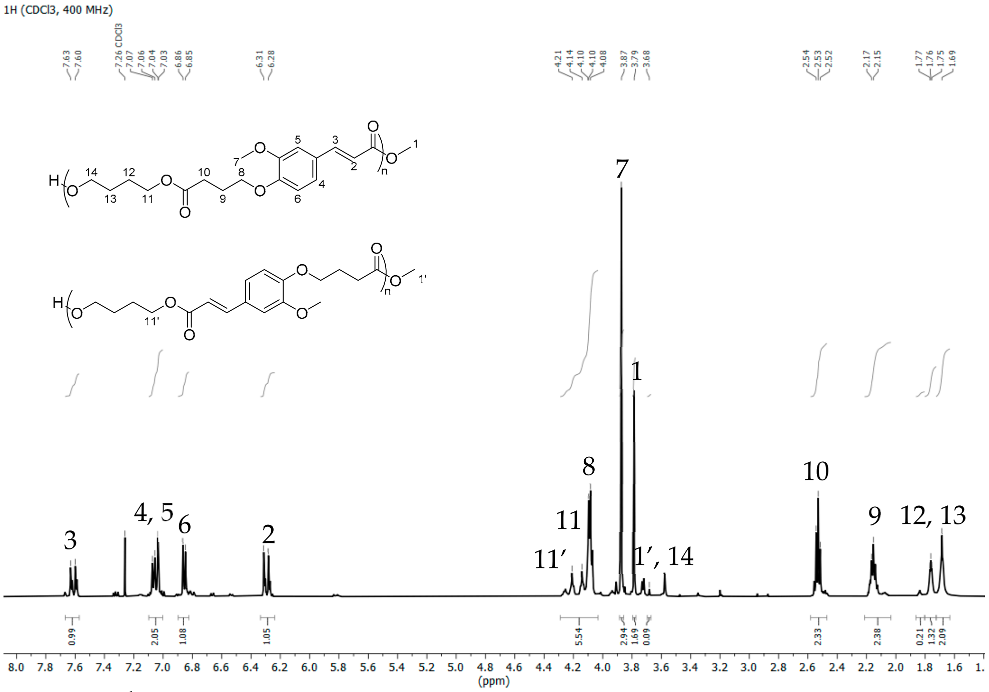

Figure S18. ^{1}H NMR analysis of poly(butylene-co-4-(methyl butanoate-oxy)-ferulate) (**3b**).

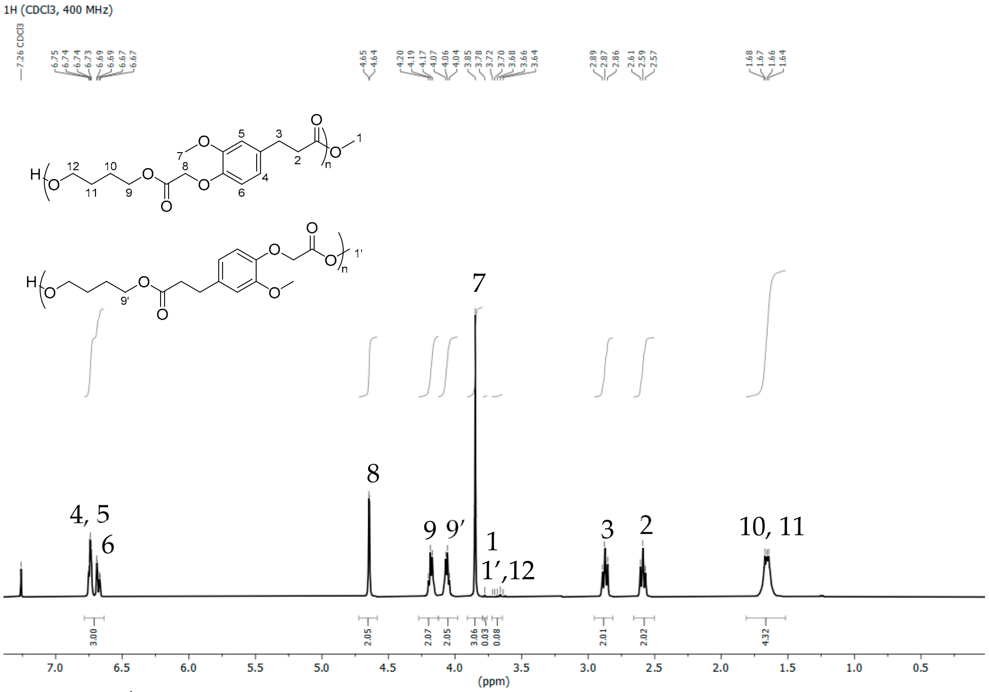

Figure S19. ^{1}H NMR analysis of poly(butylene-co-3-(3-methoxy-4-(2-methoxy-2-oxoethoxy)phenyl)propanoate) (**5a**).

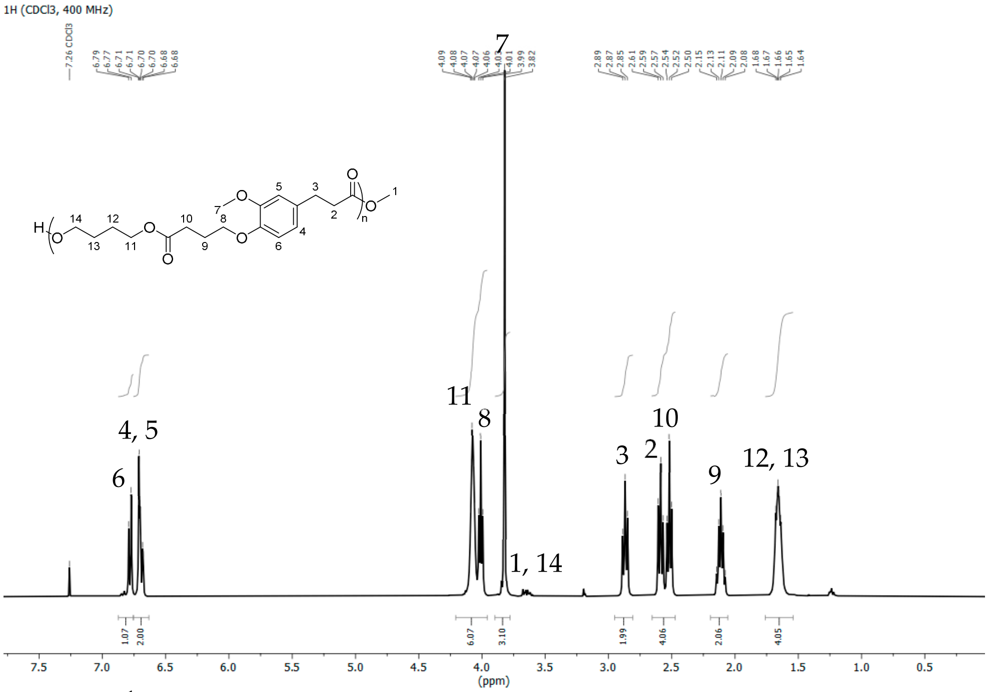

Figure S20. ^{1}H NMR analysis of poly(butylene-co-4-(2-methoxy-4-(3-methoxy-3-oxopropyl)phenoxy)butanoate) (**5b**).

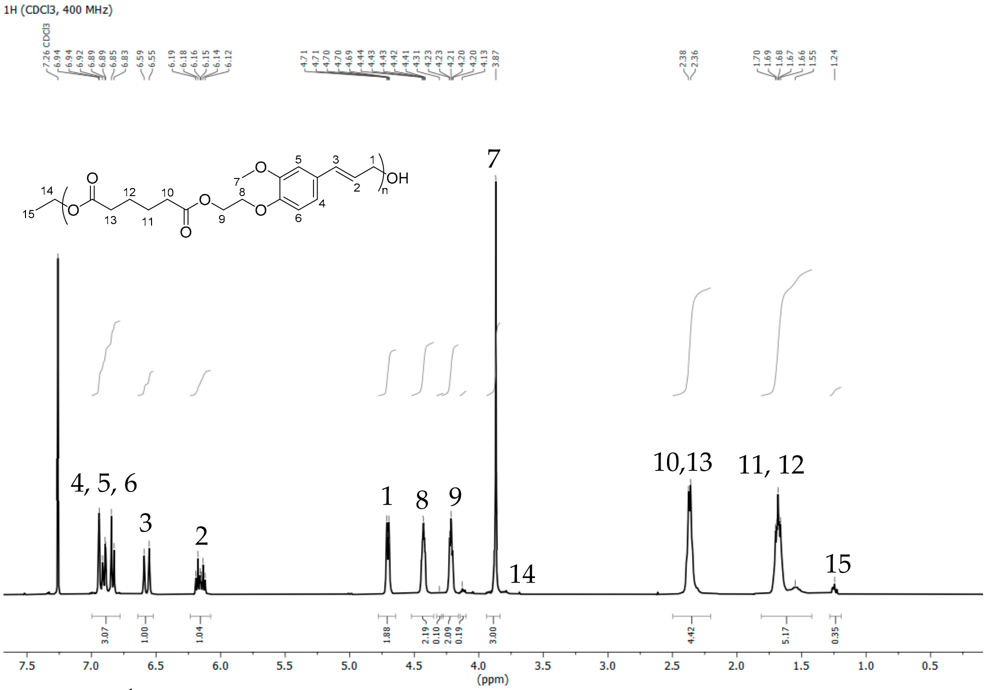

Figure S21. ^{1}H NMR analysis of poly(4-(hydroxyethoxy)-coniferylene -co-adipate) (**7a**).

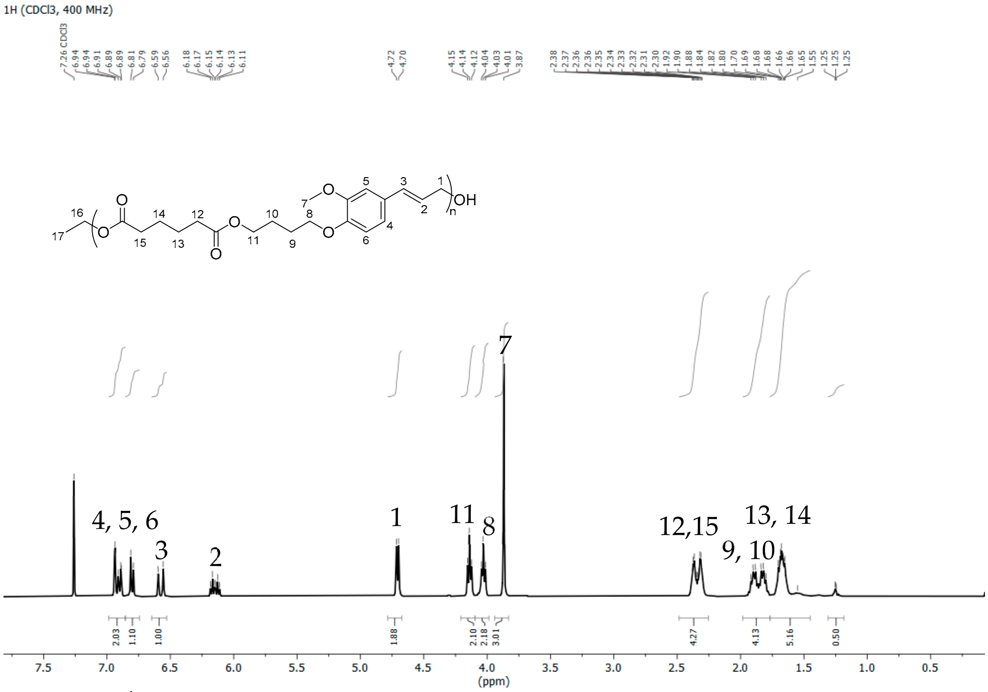

Figure S22. ^{1}H NMR analysis of poly(4-(hydroxybutoxy)-coniferylene-co-adipate) (**7b**).

5. SEC analyses of the polyesters

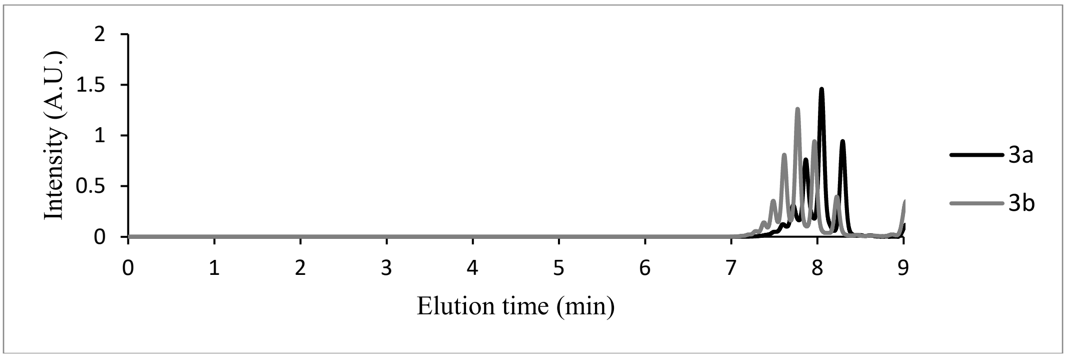

Figure S23. SEC analyses in THF of the ferulic diester-based polyesters **3a** and **3b** (curves were normalised by their area).

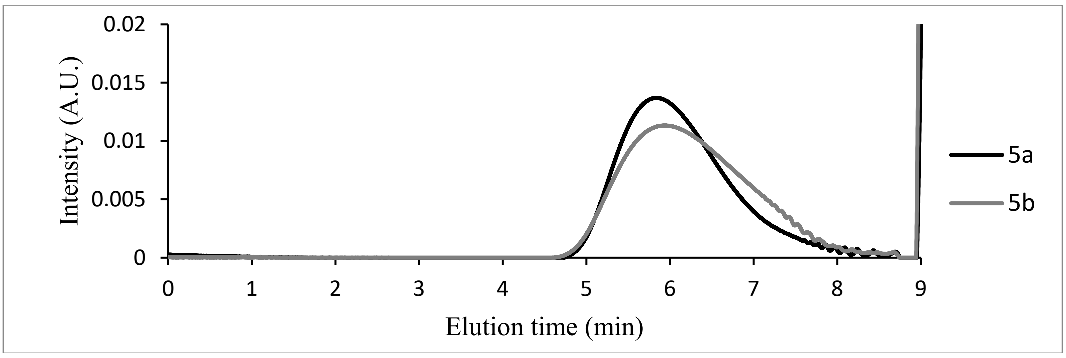

Figure S24. SEC analyses in THF of the hydrogenated ferulic diester-based polyesters **5a** and **5b** (curves were normalised by their area).

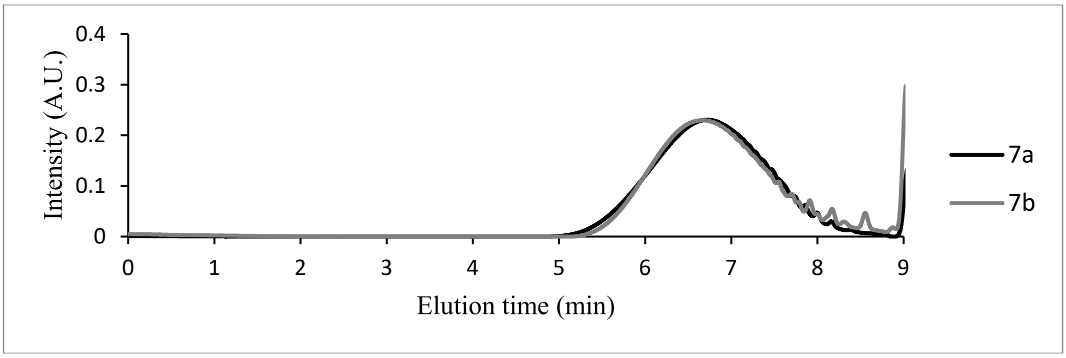

Figure S25. SEC analyses in THF of ferulic diol-based polyesters **7a** and **7b** (curves were normalised by their area).

6. Polyesters synthesized from ferulic diol in excess

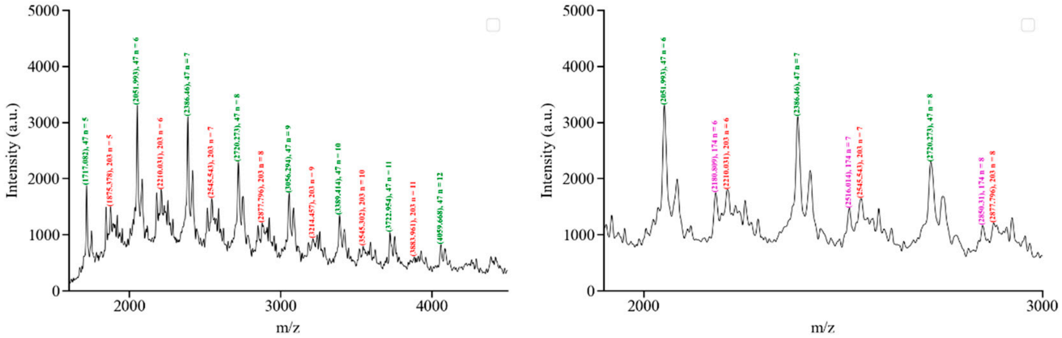

Figure S26. MALDI-TOF MS analysis of 7a obtained with 1.1 equivalent of diol in comparison to the diester after 24 hours of synthesis in acetophenone at 90°C and under reduced pressure.

Table S1. Mass of the corresponding end-groups in MALDI-TOF MS analysis of **7a** obtained with 1.1 equivalent of diol in comparison to the diester after 24 hours of synthesis in acetophenone at 90°C and under reduced pressure.

Structure	End-group description	Residual mass (m/z)
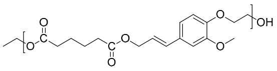	Ester and OH	46
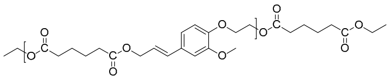	Both ester	202
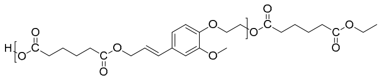	Hydrolysis	174

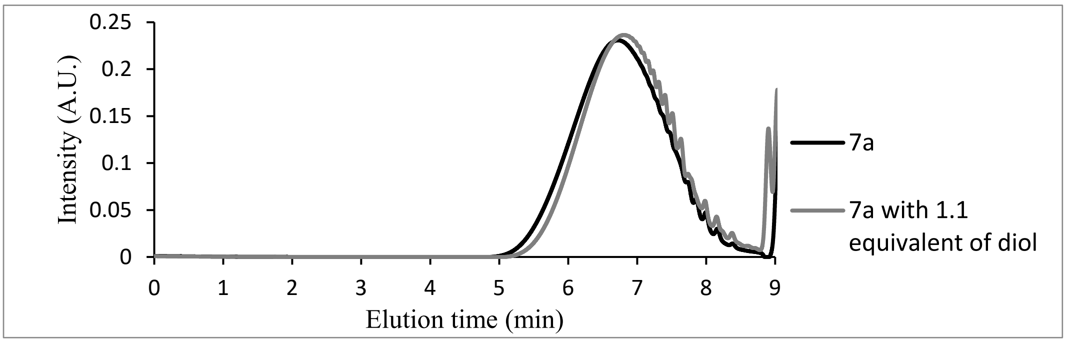

Figure S27. SEC analyses in THF of ferulic diol-based polyesters **7a** synthesized with and without an excess of diol (curves were normalised by their area).

Table S2. Average molar mass and dispersity from the SEC analyses in THF of ferulic diol-based polyesters **7a** synthesized with and without an excess of diol.

Sample name	M_n (g.mol^{-1})	M_w (g.mol^{-1})	Đ
7a	6515	14970	2.298
7a with 1.1 equivalent of diol	5345	12528	2.344
